# Velocity-dependent heat transfer controls temperature in fracture networks

**DOI:** 10.1038/s41467-023-36034-w

**Published:** 2023-01-23

**Authors:** Thomas Heinze, Nicola Pastore

**Affiliations:** 1 Department of Hydrogeochemistry and Hydrogeology; Institute of Geology, Mineralogy and Geophysics, Ruhr-University Bochum, Universitaetsstr. 150, 44801 Bochum, Germany; 2 DICATECh Department of Civil, Environmental, Building Engineering, and Chemistry, Politecnico di Bari, Via Edoardo Orabona 4, 70125 Bari, Italy

**Keywords:** Hydrogeology, Hydrology, Volcanology

## Abstract

Heat transfer between a fluid and the surrounding rock in the subsurface is a crucial process not only, but most obviously, in geothermal systems. Heat transfer is described by Newton’s law of cooling, relating the heat transferred to a coefficient, the specific surface area, and the temperature difference between rock and fluid. However, parameterizing the heat transfer coefficient in fracture networks poses a major challenge. Here we show that within a fracture network the heat transfer coefficient is strongly heterogeneous but that laboratory single fracture experiments can provide a reasonable estimate in dependence of flow rate. We investigate the distribution of the heat transfer coefficient experimentally as well as numerically and analyze the heat transfer at individual fractures. Our results improve the prediction of temperatures in engineered and natural geothermal systems and allow sustainable management and design of reservoirs considering the role of individual fractures.

## Abstract (web summary)

Heat transfer in a fracture network is heterogeneous as it depends pre-dominantly on flow velocity and fracture aperture. This finding has direct implications for the heat distribution and exploitation in geothermal and associated natural systems.

## Introduction

Rock fractures provide preferential fluid pathways due to their lower hydraulic resistance compared to the surrounding host rock and transport most of the thermal energy^1^. Especially systems with a very heterogeneous distribution of permeability and porosity, such as fractures within a low-permeable host rock, are known to experience substantial local thermal non-equilibrium (LTNE) effects so that the simplifying assumption of local thermal equilibrium (LTE) cannot be applied and heat transfer between phases needs to be explicitly described^2,3^. The heat transfer coefficient is known to depend on flow velocity and fracture aperture, while other factors such as fracture surface morphology probably are of minor effect^4–7^. Fracture surface morphology might become more relevant for heat extraction with decreasing fracture apertures^8^. Common theoretical approaches, such as the thermal boundary layer theory, overestimate the heat transfer coefficient as the width of rock fractures is usually too small for such a boundary layer to develop^9^. So far, experiments and numerical as well as theoretical works focused on single fractures due to their geometrical simplicity^7,8,10^.

Understanding heat transfer and heat transport in a fracture network are crucial to predict possible heat extraction, optimizing the heat extraction strategy to delay thermal breakthrough, and for managing reservoir conditions with respect to dynamic changes such as clogging of fractures due to chemical precipitation. However, so far existing studies did not study the interplay of fractures in a network with respect to heat transfer because a suitable model as well as experimental data for comparison were lacking^8^. Due to this knowledge gap, current approaches applied a range of parameters for the heat transfer coefficient from 20 W/(m^2^ °C) to 3000 W/(m^2^ °C) identifying a significant difference in predicted time of thermal breakthrough and production temperature at a reservoir scale^11,12^.

Here, we present a mathematical and numerical framework to describe heat transfer processes in fracture networks and compare our results to a unique set of bench-scale experiments. Using additional simulations, we investigate the sensitivity of outflow temperature with respect to heat transfer along individual flow paths in the network. Heterogeneous heat transfer based on the diverse flow field in the fracture network is crucial to explain the experimental results and amplifies heat extraction along hydraulically dominating fractures.

## Results

### Bench-scale fracture network

**Fig. 1:**
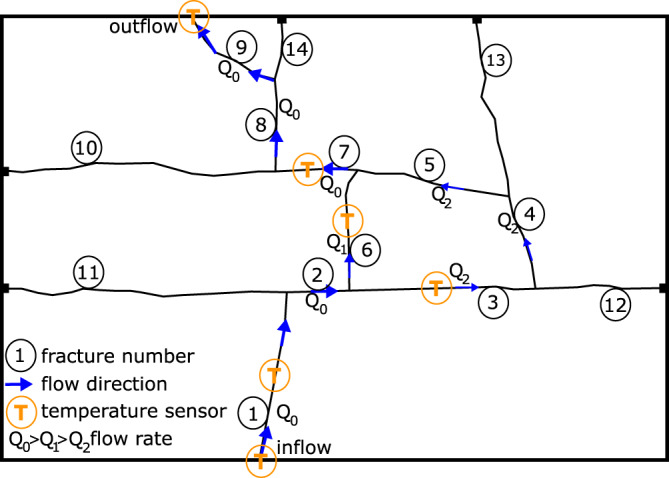
Experimental setup. Fracture network of the bench-scale experiments with fractures 1 to 9 contributing to flow and fractures 10 to 14 with dead ends. The total flow rate *Q*_0_ is split into *Q*_1_ and *Q*_2_ for the two parallel flow paths through fracture 6 and fractures 3 to 5. Water temperature is recorded at in- and outflow. Rock temperature is recorded in fractures 1, 3, 6 and 7.

The experimental setup is a limestone block of 8 cm × 40 cm × 60 cm intermitted by 14 fractures from which 9 contribute to water flow through the system with varying degrees, while 5 fractures have dead ends (Fig. 1). The rock temperature is around 15 °C and the fracture network is saturated by water of similar temperature prior to the experiment. The experiments start with the injection of warm fluid with peak temperatures between 45 °C to 50 °C with a pulse duration of approximately 1000 s. Water temperature is measured at in- and outflow and rock temperature at 4 points in the system in the middle of the fractures. Flow rate *Q*_0_ is varied 21 times between 1 × 10^−6^m^3^/s to 1 × 10^−5^m^3^/s (see Supplementary Table 1).

The water flows through fractures 1, 2, 7, 8, and 9 with rate *Q*_0_ and there is no flow through fractures 10 to 14 due to their dead ends. The flow rate *Q*_0_ is split in flow rate *Q*_1_ flowing in fracture 6 and flow rate *Q*_2_ flowing through fractures 3, 4 and 5 (Fig. 1). The fluxes *Q*_1_ and *Q*_2_ are calculated using an explicit network model depicting each fracture as a one-dimensional pipe element^13^. In previous works, flow and mass transport experiments were used to estimate the flow rate and water velocity which crosses every single fracture^13,14^. The values for the Forchheimer equation with constant terms for the whole fracture network obtained from these previous studies were used to calculate the flow resistance and the flow rate in the branches of the fracture network. At the intersection of fractures 2, 3 and 6, fracture 6 is taking most of the total flow rate due to its lower hydraulic resistance, resulting in *Q*_1_ > *Q*_2_^13^. Additional experimental details are provided in the Methods section and calculated values for *Q*_1_ and *Q*_2_ are presented in Supplementary Table 1. A sketch of the experimental setup is shown in Supplementary Fig. 1.

The numerical reproduction of these experiments is based on a continuous rock body that interacts with the fluid flowing in the fractures through an explicit heat transfer term. Heat transport in the rock is limited to conduction, while the heat inside the fractures is subjected to advective and conductive transport. Heat between the solid rock and the water in the fractures is exchanged based on Newton’s law of cooling

1 $$Q=hA\Delta T$$

with the heat transfer coefficient *h* (W/(m^2^ °C)), specific surface area *A* (1/m), and the temperature difference between rock and water Δ*T* (°C). As the phase temperatures, governed by Eqs. (2) and (3), change over time due to the inflow of cold water, the subsequent heat transport processes, and heat exchange, the temperature difference Δ*T* also changes over time. At the confluence of fractures 5 and 6, the thermal energy is weighted by the mass flow through the respective fracture assuming perfect mixing. The heat transfer coefficient is adjusted in all fractures to achieve the best match between numerical simulation results and the experimental observation. To further constrain the heat transfer coefficient, sets of fractures were generated based on the respective flow velocities for which a common heat transfer coefficient was assumed. The first set consists of fractures 1, 2, 7, 8, and 9 with flow rate *Q*_0_, the second set is fractures 3 to 5 with flow rate *Q*_2_, fracture 6 is a separate case with flow rate *Q*_1_, and the last set includes fractures 10 to 14 with no water flow. In fractures 10 to 14 the heat transfer coefficient is kept constant for all experiments, as these fractures are not affected by varying the total discharge rate *Q*_0_. The heat transfer coefficient for the other sets of fractures is the only parameter adjusted for each simulation to achieve the best possible agreement between temperatures measured in the experiments and numerical simulation results. The numerical implementation is described in more detail in the Methods section.

### Numerical simulation of experiments

**Fig. 2:**
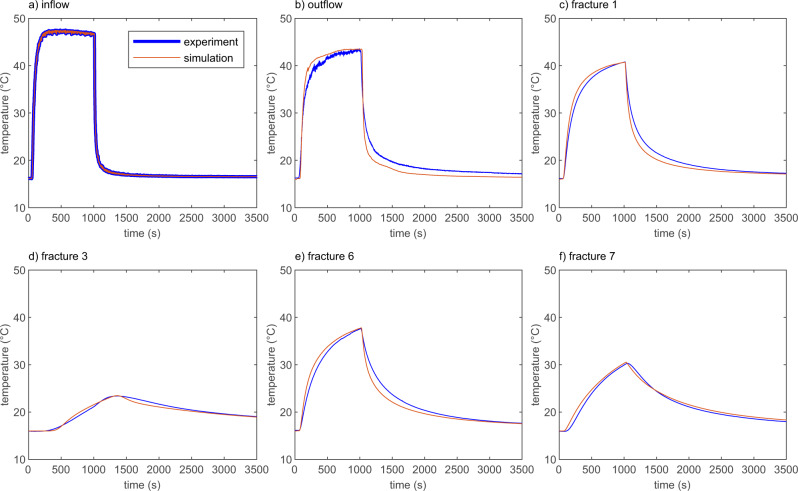
Thermal breakthrough curves. Water temperature at in- (**a**) and outflow (**b**) and rock temperatures recorded in the middle of fracture 1 (**c**), fracture 3 (**d**), fracture 6 (**e**), and fracture 7 (**f**) for a flow rate of 7.9 × 10^−6^ m^3^/s during the experiment and the numerical simulation. Difference between best-fit simulations and experimental data is less than 1 °C for the vast majority of the recorded 126 TBTC (Supplementary Figs. 2 to 22) besides few exceptions.

Six thermocouples record the thermal break-through curves (TBTC) at in- and outflow as well as in the middle of the fractures 1, 3, 6, and 7 (Fig. 2). The shape of the injected temperature pulse smears along its way through the fracture network, from an originally almost rectangular shape (Fig. 2a) to a more and more rounded shape with decreasing amplitude in fractures 1, 6, and 7 (Fig. 2c, e, f). The TBTC in fracture 3 has a less rapid incline, reaches its peak temperature later than the other TBTCs and the temperature drop is slower compared to other fractures (Fig. 2d). While the sensors at the fractures are obtaining rock temperatures, inflow and outflow are water temperatures. Therefore, the outflow temperature can be higher than the rock temperatures measured along the flow path. The agreement between experimentally measured (blue curve in Fig. 2) and numerically calculated temperatures (red curves in Fig. 2) at all 6 temperature sensors for all 21 discharge rates, resulting in 126 TBTC, is within a range of 1 ^∘^C (max. relative error of 2.5 % besides very few exceptions (Fig. 2 and Supplementary Figs. 2 to 22).

The values of the heat transfer coefficient *h* of individual rock fractures used in the numerical simulations providing the best fit to the experimental data are in the range of 1 W/(m^2^ °C) to 260 W/(m^2^ °C) (Supplementary Fig. 23). A sensitivity study of the heat transfer coefficient shows that the resulting temperature distribution is sensitive to the value of *h* in a single fracture within a range of 80% to 120% of its original value causing different shapes of the TBTC as well as peak temperatures (Supplementary Fig. 24). Consequently, simple variations of the numerical model, such as setting the heat transfer coefficient homogeneous across all fractures even for a homogeneous flow velocity, cause a systematic disagreement between experimental and numerical results.

The fractures 1, 2, 7, 8, and 9 with the highest flow rate *Q*_0_ have the highest heat transfer coefficient. Fracture 6 with flow rate *Q*_1_ < *Q*_0_ has an intermediate value of *h*, while fractures 3 to 5 with flow rate *Q*_2_ < *Q*_1_ have the lowest heat transfer coefficient values (Supplementary Fig. 24). In all fractures the values of *h* increase with increasing flow rate. Small disturbances can be observed at very low flow rates, possibly due to unsteady flow in the network and related variation in the relevant heat transfer area in rough fractures^15^. The values obtained from the best fit cover a similar value range and tendency than the ones predicted using the newly presented empirical equation (Supplementary Fig. 24). All curves seem to approach a saturation value for high flow rates. Such behavior is quite well-known from single fracture experiments^4,16^ and is therefore also part of the newly derived empirical model. The effect can be explained through the thermal boundary layer theory and its inverse dependence on the square root of the flow velocity. However, rock fractures are usually considered to narrow for the evolution of a thermal boundary layer^9^ and recent experiments indicate a counteracting effect at flow rates above 0.2 m/s flow velocity in a single rock fracture with 1.2 × 10^−4^ m aperture^17^.

**Fig. 3:**
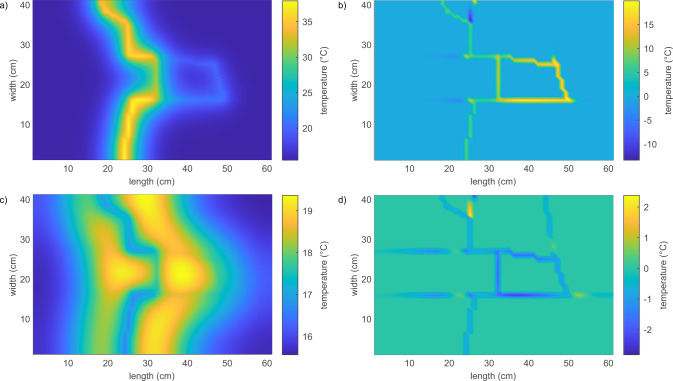
Temperature distribution. Calculated rock temperatures (**a**, **c**) and difference between water and rock temperatures (**b**, **d**) for snapshots at 1000 s (**a**, **b**), close to the end of the injected temperature pulse, and 3000 s (**c**, **d**) after the start of the simulation for a flow rate of 7.9 × 10^−6^ m^3^/s. A temperature difference of several degrees Celsius between water and rock is sustained during the experiment.

As a pulse injection is considered, the heat is initially transferred from the injected warm water into the host rock. The rock temperature increases more strongly around the fractures 1, 2, 6, 7, 8, and 9 with the higher flow rates *Q*_0_ and *Q*_1_ than around fractures 3 to 5 with flow rate *Q*_2_. This can be seen from the experimentally obtained rock temperatures around fractures 1, 3, 6, and 7 (Fig. 2c–f), as well as from the calculated distribution of rock temperature using the corresponding numerical simulation (Fig. 3a) and the calculated TBTC (Supplementary Figs. 2–22). The maximum temperature increase around fractures 1, 2, 6, 7, 8, and 9 is slightly below 40 °C after 1000 s (Figs. 3a and 2c). The temperature increase around fractures 3 to 5 is much smaller with rock temperatures around 23 °C (Fig. 3a and Fig. 2d). The temperature increase around fractures without flow is below 2 °C (Fig. 3a). However, due to conduction, the rock can warm around fractures with no active fluid flow but heat is barely transferred to the water (e.g., fracture 14 in Fig. 3a and b). The temporal evolution is shown in Supplementary Figs. 2–22. The calculated temperature difference between rock and water is up to 17 °C at the end of the temperature pulse after approximately 1000 s (Fig. 3b). After the end of the temperature pulse, the temperature of the incoming water temperature drops rapidly to its initial value, 16 °C in the experiment shown in Fig. 2a. However, the rock got heated during the warm water pulse and is therefore warmer than the water flowing through the fractures, reverting heat transfer. The rock can sustain its elevated temperatures for several hundreds of seconds, especially in areas with small water flux (Fig. 2d). Water outflow temperature is also higher than the inflow water temperature for several hundreds of seconds (Fig. 2b). The numerical simulations support this interpretation as calculated rock temperatures are warmer than the water temperatures but the heating effect is smaller than during the warm water pulse as temperature differences between phases are around 2 °C (Fig. 3c, d).

### Thermal breakthrough simulations at bench-scale

For further investigation, additional simulations of a reversed heat transfer with an initial rock temperature of 45 ^°^C and a steady inflow water temperature of 15 °C are conducted. Heat transfer and hydraulic parameters were chosen similarly to the experiments with a flow rate of 2.8004 × 10^−6^m^3^/s. We study three scenarios: (1) mass flows *Q*_1_ > *Q*_2_ and heat transfer coefficients ${h}_{3-5}=15\,{{{{{{{\rm{W}}}}}}}}/({{{{{{{{\rm{m}}}}}}}}{}^{2}}^{\circ }\!{{{{{{{\rm{C}}}}}}}})$ and ${h}_{6}=50\,{{{{{{{\rm{W}}}}}}}}/({{{{{{{{\rm{m}}}}}}}}{}^{2}}^{\circ }{{{{{{{\rm{\!C}}}}}}}})$ of fracture 6 and fractures 3 to 5 remain as for the experiments above. (2) the mass flows *Q*_1_ = 2.188 × 10^6^ m^3^/s in fracture 6 and *Q*_2_ = 0.683 × 10^6^ m^3^/s in fractures 3 to 5 is reversed, so that now *Q*_1_ < *Q*_2_ with *Q*_1_ = 0.683 × 10^6^ m^3^/s and *Q*_2_ = 2.188 × 10^6^ m^3^/s but the heat transfer coefficients are not altered (${h}_{3-5}=15\,{{{{{{{\rm{W}}}}}}}}/({{{{{{{{\rm{m}}}}}}}}{}^{2}}^{\circ }\!{{{{{{{\rm{C}}}}}}}})$ and ${h}_{6}=50\,{{{{{{{\rm{W}}}}}}}}/({{{{{{{{\rm{m}}}}}}}}{}^{2}}^{\circ }{{{{{{{\rm{\!C}}}}}}}})$). (3) additionally to the change in mass flow ratios also the heat transfer coefficients are switched (${h}_{3-5}=50\,{{{{{{{\rm{W}}}}}}}}/({{{{{{{{\rm{m}}}}}}}}{}^{2}}^{\circ }{{{{{{{\rm{C}}}}}}}})$ and ${h}_{6}=15\,{{{{{{{\rm{W}}}}}}}}/({{{{{{{{\rm{m}}}}}}}}{}^{2}}^{\circ }{{{{{{{\rm{\!C}}}}}}}})$). The scenarios are chosen to investigate the impact of the velocity-dependent heat transfer coefficient independently of the actual mass flow.

**Fig. 4:**
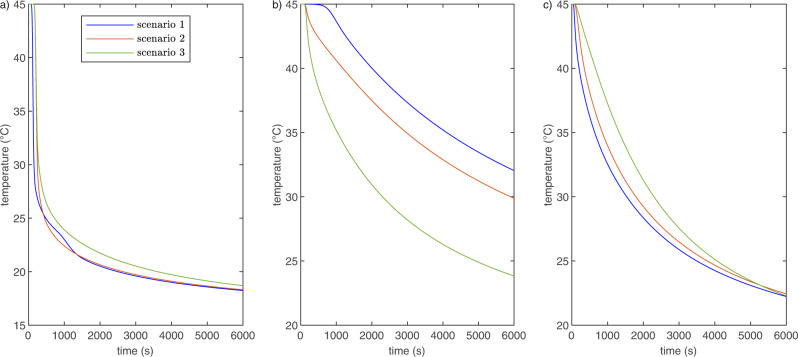
Scenario testing. Simulated water temperatures at outflow (**a**) as well as rock temperatures at fractures 3 (**b**) and 6 (**c**) for a water inflow of 15 °C in the bench-scale setup with a rock temperature of 45 °C. Scenarios: (1) mass flow according to the explicit network model with matching *h* values. (2) reversed mass flow rated between fractures 3 to 5 and 6 but unaltered *h* values. (3) reversed mass flow and matching *h* values.

The shift in mass flow ratios (scenarios 2 and 3), effectively guiding the water flow through fractures 3 to 5 instead of fracture 6, delays the thermal breakthrough time (drop in outflow temperature by 1 °C) from 100 s in scenario 1 to 180 s for scenario 3 (Fig. 4a). While the time of thermal breakthrough is less than a few seconds different between scenarios 2 and 3, the drop in outflow temperature is way more rapid in scenario 2 than in scenario 3. Accordingly, the drop in temperature in fracture 3 is up to 9 °C (after around 4000 s) larger in scenario 3 than in the other scenarios (Fig. 4b). In fracture 6, the difference of temperature evolution for the different scenarios is around 5 °C after approximately 500 s (Fig. 4c).

### Reservoir-scale fracture network

**Fig. 5:**
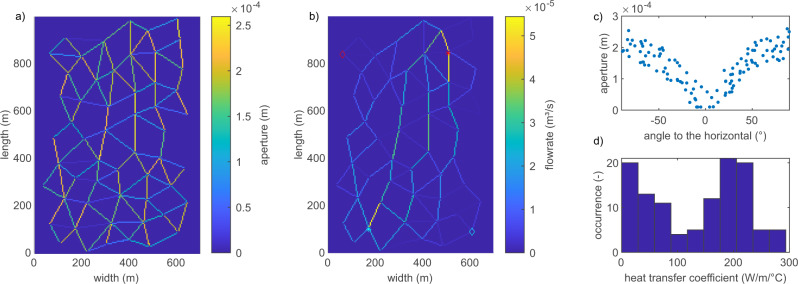
Numerical reservoir. Fracture network of the numerical geothermal reservoir with 116 fractures. The aperture (**a**) is calculated based on an assumed stress field oriented roughly north to south. Line thickness is enhanced for visibility. Injection (red) and production borehole (blue) are marked for two well configurations: 1 (asterisks) and 2 (diamonds). The flow rate is maximum along preferential flow paths within the network (**b**). Aperture varies with fracture orientation within the stress field (**c**). Distribution of resulting heat transfer coefficients (**d**).

For upscaling to reservoir scale, we study a hypothetical geothermal reservoir derived from outcrop data of Whitby Mudstone along the Yorkshire coast (UK)^18^. Whitby Mudstone is the exposed counterpart to Posidonia Shales present in a potential unconventional gas reservoir in the Netherlands^18^. The outcrop data has been scaled up to cover a range of approximately 700–1000 m and used for geothermal reservoir simulations in the past^19^. In the reservoir, fractures are the dominant flow paths in the system due to the low intrinsic permeability and porosity of the Mudstone varying between 1 × 10^−18^ m^2^ to 1 × 10^−21^ m^2^ and 0.5% to 2.5%, respectively^18^. The accurate representation of fracture networks is subjected to required simplifications while preserving the network characteristics. In this work, we compare three representations of the network with 34, 66 and 116 fractures^19^. Therefore, all networks share a mutual geometry but fracture number and fracture length vary. Further analysis of the fracture networks is presented in Supplementary Figs. 25–36. Fracture aperture varies between 1 × 10^−5^ m to 1 × 10^−4^ m depending on fracture orientation to the horizontal simulating a stress field with maximum compressive stress oriented North to South superimposed with statistical noise (Fig. 5a and c). Locations of production and injection boreholes are chosen to achieve the longest possible flow distance in the diagonal direction while providing sufficient connection to the network (Fig. 5b)^19^. The calculated apertures in combination with an injection pressure of 50 MPa result in flow velocities in the range of 1 × 10^−4^ m/s to 3 × 10^−1^ m/s in the individual fractures. Assuming an initial reservoir temperature of 150 °C with an injection temperature of 30 °C the heat distribution in the reservoir is calculated for 30 years of production.

The numerical solution of the heat transfer and transport processes is conducted similarly to the simulations of the bench-scale experiments as outlined in the Methods section. The steady-state flow field is calculated based on mass conservation in the network neglecting any possible matrix flow. At intersections of multiple fractures, thermal energy is weighted by the mass flow through the respective fractures assuming perfect mixing. The heat transfer coefficient of the individual fractures is determined in dependence on velocity and aperture using a newly derived empirical relationship presented in the Methods section based on over 240 previously published experiments for the adequate range of velocity and aperture values^4,16,20^. The resulting values of the heat transfer coefficient *h* are presented in Fig. 5d. A graphical representation of the derived relationship between heat transfer coefficient, aperture, and flow velocity is shown in Supplementary Fig. 37. For comparison simulations with various homogeneous values of the heat transfer coefficient are also presented.

### Numerical simulation of reservoir production

**Fig. 6:**
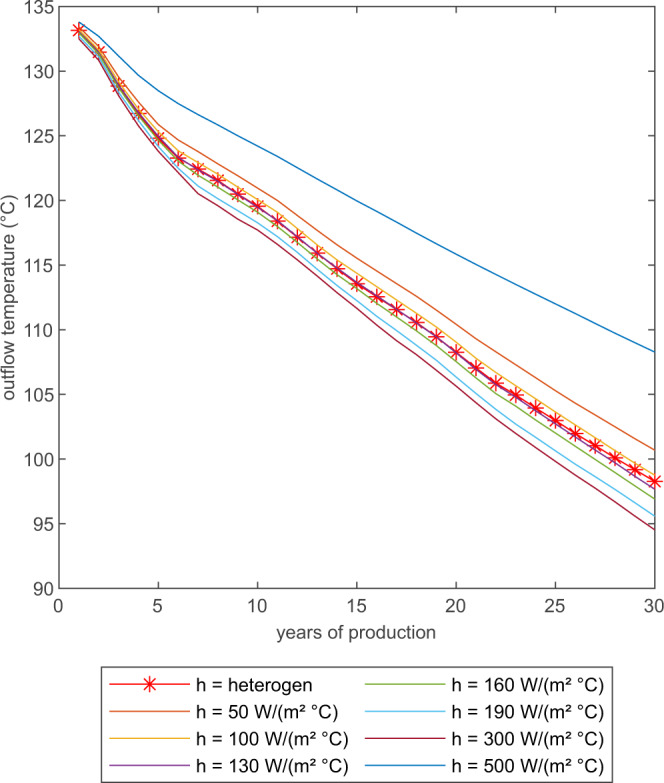
Predicted outflow temperature. Simulated water temperatures at outflow for the network consisting of 116 fractures for various values of the heat transfer coefficient *h* equal for all fractures and the heterogeneous velocity-dependent approach.

The most critical parameter for any production of geothermal energy is the outflow temperature at the production well. A precise prediction of the outflow temperature is crucial for the economic feasibility of heat mining projects. The outflow temperature depends on several parameters, such as network connectivity and flow rate, but also on the heat transfer from the host rock to the fluid flowing in the fractures (Fig. 6). The results for the other two fracture networks are shown in Supplementary Figs. 38–43. Depending on the heat transfer coefficient, outflow temperature can vary by more than 10 °C after 30 years. The outflow temperature becomes cooler for higher heat transfer coefficients because the heat transferred to the fluid exceeds the capability of the host rock to transfer heat by conduction from regions further away from the fractures towards the heat transfer interface causing a quick local cooling of the host rock around fractures and limiting its capability to sustain high fluid temperatures^11^. In general, as higher the value of the heat transfer coefficient, as less prominent the changes in outflow temperature become. With increasing values of the heat transfer coefficient, thermal equilibrium between phases becomes more likely. Still, the phase temperatures between rock and fluid persist over the whole simulation period of 30 years never reaching local thermal equilibrium between phases for all tested scenarios (Supplementary Figs. 44–46). The temperature difference between phases becomes less over the years but remains at around 70 °C for the different tested setups after 30 years. This shows that the heat transport within the rock is the limiting factor for heat extraction and not the heat transfer across the fracture area. A reasonable value range covered by the experimental data of single fracture experiments with heat transfer coefficients is between 100 W/(m^2^ °C) to 190 W/(m^2^ °C)^10,16,20^. Within this range, three simulations were conducted with heat transfer coefficients of 130 W/(m^2^ °C), 160 W/(m^2^ °C) and 190 W/(m^2^ °C). These values represent a range of 80% to 120% around 160 W/(m^2^ °C). Within this range, a variation in outflow temperature of around 2 °C can be observed (Fig. 6). This shows, how sensitive outflow temperatures at the field scale react to values of the heat transfer coefficient.

**Fig. 7:**
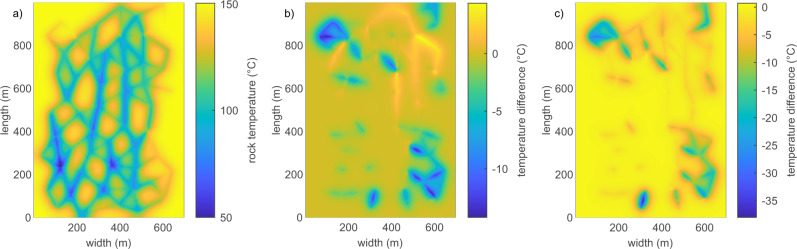
Temperature distribution. Rock temperature for the velocity-dependent heat transfer (**a**) and differences to this distribution for $h=100\,{{{{{{{\rm{W}}}}}}}}/({{{{{{{{\rm{m}}}}}}}}{}^{2}}^{\circ }{{{{{{{\rm{C}}}}}}}})$ (**b**) and $h=190\,{{{{{{{\rm{W}}}}}}}}/({{{{{{{{\rm{m}}}}}}}}{}^{2}}^{\circ }{{{{{{{\rm{C}}}}}}}})$ (**c**) in the reservoir consisting of 116 fractures after 30 years of production. Rock temperature significantly differs around individual fractures as well as for whole sections of the reservoir.

Most prominently, the rock temperature distribution within the reservoir significantly depends on the heat transfer coefficient. Considering a flow rate and temperature-dependent heat transfer coefficient, heat extraction is focused around flow-dominant fractures (Fig. 7). On the other hand, in a homogeneous distribution of heat transfer coefficients within a reservoir, the drop in host rock temperature is larger with increasing heat transfer coefficients in regions of comparably low flow rates. Heat transfer capabilities of the individual fractures control the temperature field. Due to the commonly derived fracture network geometry of the tested reservoir representations of the same areas of the reservoir are affected by the cooling (see Supplementary Figs. 42 and 43). In general, the absolute differences in the temperature field of the reservoir between models using homogeneous and heterogeneous heat transfer coefficients become larger for networks with fewer fractures, and larger areas of the reservoir are affected. As an example, in all networks, the north-west corner of the reservoir is barely affected in the heterogeneous heat transfer model but cools by more than 20 °C after 30 years for homogeneous distributions of the heat transfer coefficient (Fig. 7).

The outflow temperature does not necessarily reflect these differences in temperature distribution in the reservoir as the mixture of fluids in a well-connected network masks local heterogeneity. The distribution of heat transfer coefficient values in the presented case (Fig. 5) peaks around 190 W/(${{{{{{{{\rm{m}}}}}}}}{}^{2}}^{\circ }$C). The calculated outflow temperature for the heterogeneous case is within the range but distinguishable different also in the dynamic changes over time by more than 1 ^∘^C from the calculations using a homogeneous *h* of 190 W/(${{{{{{{{\rm{m}}}}}}}}{}^{2}}^{\circ }$C) (Fig. 6). This is caused by the differences in local temperature distributions in the reservoir and the differences in the heat transfer capabilities of individual fractures.

### Influence on reservoir design and management

**Fig. 8:**
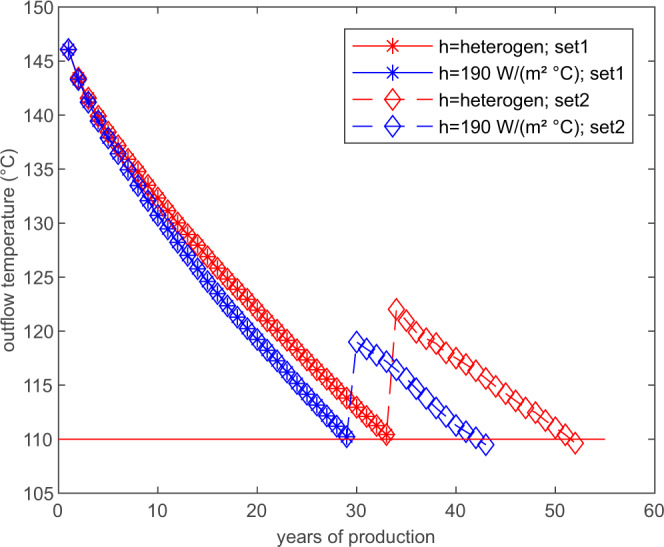
Switch in well configuration. Simulated water temperatures at outflow for a switch from well configuration 1 to configuration 2 once a threshold of 110 °C is reached. The heterogeneous heat transfer simulation results in 10 years more of reservoir lifetime.

Describing heat transfer in a fracture network so far was hindered by two important aspects: (i) the assumption of a homogeneous heat transfer, and (ii) the choice of a suitable value of the heat transfer coefficient. Both issues are addressed in this work. To demonstrate the influence of the presented work on design and management of geothermal systems, the setup above is repeated with a lower injection pressure of 30 MPa. This lower injection pressure affects the distribution of the heat transfer coefficient in the reservoir but values of the heat transfer coefficient still peak around 190 W/(${{{{{{{{\rm{m}}}}}}}}{}^{2}}^{\circ }$C) (Supplementary Fig. 47), so that the value of 190 W/(${{{{{{{{\rm{m}}}}}}}}{}^{2}}^{\circ }$C) is still a reasonably good estimate for a homogeneous heat transfer coefficient for comparison. Further, it is a known problem in geothermal energy production that parts of the reservoir remain poorly activated and well placement is a crucial parameter to minimize this loss^21,22^. To demonstrate the effect of velocity-dependent heat transfer in fractures on reservoir design and well placement, we switch to borehole configuration 2 once the production temperature drops below 110 °C, a reasonable limit for electric power generation from geothermal energy (Fig. 5). With configuration 2 regions barely affected by the original flow field get utilized for heat mining. With this switch in borehole locations, the difference in production temperature between conventional predictions with constant heat transfer coefficient and simulations with a heterogeneous, velocity-dependent heat transfer become larger over time and an increase in the production time of the reservoir is observed (Fig. 8). The temperature difference between homogeneous and heterogeneous heat transfer simulation after 29 years, when the homogeneous heat transfer simulation reaches the 110 °C threshold, is 3.6 °C. This temperature difference results in a difference of 4 years of estimated run-time for the reservoir based on well configuration 1. Including the switch to well configuration 2, the heterogeneous heat transfer simulation shows higher outflow temperatures and the estimated run-time is 10 years longer than for the homogeneous heat transfer calculation. During the use of configuration 2, the rock temperature around the wells of configuration 1 and major flow paths of this configuration recovered up to 30 °C (Supplementary Fig. 48), providing the possibility of further prolonging the reservoir lifetime by switching back to configuration 1.

## Discussion

The lifetime and sustainability of a geothermal reservoir are critical for its return on financial investment. As drilling and subsurface engineering account for 30% to 50% of the total investment costs^23^ a simple doublet system is often chosen besides known benefits of multi-well layouts^24,25^. We show that through the consideration of heterogeneous heat transfer the installation of additional wells might be economically efficient, as we calculated an extended lifetime of 10 years compared to conventional estimations (Fig. 8). The additional wells activate previously unused parts of the reservoir and the additional gain in extracted heat is substantially larger than previously thought. While previously unused parts of the reservoir are stimulated, the cooled parts of the reservoir can thermally recover. This provides chances for prolonging the lifetime of geothermal plants even further. The proposed addition of wells can be applied to existing geothermal plants, as well as to future geothermal systems. Additionally, from an economic point of view, the second set of boreholes can be drilled substantially after the start of the reservoir production. This provides time to investigate the reservoir under production conditions to find the most suitable position for the well locations. Also, the addition of just one additional well in combination with the existing doublet might be economically reasonable.

While these calculations and the economic return are reservoir-dependent, our approach is rather conservative, as multiple factors that would increase the difference between heterogeneous and homogeneous heat transfer calculations have been neglected. As such, non-linear flow around the injection well might cause higher flow velocities and therefore intensify heterogeneous heat transfer. Further, many thermally triggered effects such as the increase in fracture apertures due to thermal compaction during cooling^26,27^, fines mobilization^28,29^, or biofilm generation^30,31^ might strongly affect a reservoir’s longtime behavior. The prediction of these effects can possibly be improved with heterogeneous heat transfer models because they might occur more locally along individual fractures than previously thought. This is also valid for earthquake prediction triggered by thermal stresses^32–36^. In our simulations, the heat transfer coefficients are considered heterogeneous but constant over time. However, the occurrence of these effects is reservoir specific and the prediction of the influence of those processes on the heat transfer, similar to reservoir clogging by mineral precipitation^37^, poses a future challenge for the prediction of longtime reservoir behavior and requires a multi-physics approach. These changes in the hydraulic behavior of a reservoir can also benefit geothermal exploitation, as flow paths inside the fracture network might become altered so that thermal exploitation in previously neglected parts of the reservoir is increased. Clogging and opening of fractures can also be triggered through anthropogenic stimulation techniques^38,39^ and might provide future optimization techniques for geothermal reservoirs.

The influence of the heat transfer coefficient on outflow temperature estimation and thermal breakthrough predictions cannot be neglected, which contradicts current state-of-the-art procedures^12,40,41^. Precise knowledge of the reservoir’s fracture network has been found essential for reservoir planning^42^ and is even more relevant based on our findings. The velocity-dependent heat transfer coefficient amplifies the contribution of hydraulically dominant fractures with the highest mass flow rates^43^ causing local temperature differences between homogeneous and velocity-dependent heat transfer of up to 30 °C (Supplementary Fig. 43). The influence of the heterogeneous heat transfer on the outflow temperature increases with a decreasing number of fractures (compare Fig. 6 and Supplementary Figs. 40 and 41). This has direct implications for the reservoir design as current approaches often favor hydraulic stimulation techniques generating single large fractures^44–46^. Instead, our simulations suggest that from a purely thermal point of view homogeneous, well-connected fracture networks with a small variation in fracture aperture are preferable for optimal heat exploitation. The generation of such networks requires either specific hydraulic stimulation procedures or different stimulation techniques. With a growing number of available chemical stimulation techniques for geothermal reservoirs, our results encourage further research^47–50^.

From its mathematical concept, the heat transfer coefficient of a fracture is independent of the length or width of the fracture, because these spatial dimensions are scaled by the heat transfer area within Newton’s law of cooling^10^. The heat transfer coefficient depends on flow velocity and aperture, possibly among other factors, which in principle are independent factors from the spatial extent of a fracture. Our results show that values of the heat transfer coefficient obtained from small-scale single fracture experiments with the size of a few centimeters apply to bench-scale experiments and provide reasonable estimates for reservoir scale.

In the future, single fracture experiments need to be extended for temperatures up to 350 ^∘^C and pressure conditions of up 80 MPa for water and CO_2_ to allow an application to all kinds of reservoir conditions, including supercritical geothermal systems^32,51^. Unification of experiments is also required. Currently, the relationship between flow velocity and heat transfer coefficient is not comparable across different experimental settings because increasing pressure gradients can also alter the flow-through area of a fracture^15^. To further bridge the scale gap, mesoscale heat transfer experiments on near-surface fracture networks might be useful to verify the findings presented in this work to a greater extent. Near-surface mesoscale fracture networks can provide a well-confined setting with more accessible monitoring methods than deep underground reservoirs.

The presented simulations support the hypothesis that the heat transfer coefficient does not change remarkably with the direction of the heat transfer. Both directions can be described within the accepted divergence with the same value of the heat transfer coefficient. This finding is in agreement with previous experimental work in single fractures^17^. The presented sensitivity study of the heat transfer coefficient *h* at laboratory and field scale is also in agreement with other hypothetical simulations of synthetic geothermal reservoirs resulting in outflow temperatures variations of up to approximately 15 °C using a homogeneously distributed heat transfer coefficient^11^. Precise predictions of outflow temperatures with a range of 1 –2 °C require an estimation of the heat transfer coefficient within a range of 20% or less (Fig. 6 and Supplementary Figs. 24, 40, and 41).

The persistence of local thermal non-equilibrium in experiments and simulations (Figs. 3 and Supplementary Figs. 44–46) questions the validity of the common LTE assumption for hydrothermal systems. We also envision the application of our findings to volcanic systems in which heterogeneous heat distributions might be explained through heterogeneous heat transfer processes^52,53^, as well as to thawing processes in fractured permafrost rock^54^.

## Methods

### Experimental procedure

The limestone was quarried at the Calcare di Altamura formation in the Apulia region in southeastern Italy. After cutting, the fracture network was generated through blows with a 5 kg hammer. The fractures have been cleaned from debris, reassembled, and the fissures on the block surfaces were sealed with silicone. Once it hardened, a thin layer of epoxy resin with a thermal conductivity of 0.502 W/(m°C) was applied with a brush on all faces of the block. Successively, a frame of 0.085 m × 0.405 m × 0.605 m was built around the block and epoxy resin was poured between the limestone and the frame to obtain a thickness of epoxy resin on all faces of 0.0025 m. At the ends of fractures hitting the end of the block, holes were drilled with a diameter of 0.01 m, closed with a hexagonal bushing, and sealed with epoxy resin. These openings allow the insertion or release of water or air acting as vents.

Temperature sensors have been placed at the inlet and outlet port as well as within the rock sample in correspondence with the rock-fracture interface through the opening of a small hole (0.002 m) sealed with rapid-hardening epoxy resin. The temperature sensors have been connected to a TC-08 Data Logger (pico Technology) with a sampling rate of 1 s. For thermal isolation, the whole setup had been surrounded with an extruded polystyrene layer of at least 5 cm thickness with a thermal conductivity of 0.034 W/(m°C). The flow rate between the upstream reservoir and outflow has been measured with an ultrasonic velocimeter (DOP3000 by Signal Processing). The water was heated using an electric boiler (Ariston 3100313) with a volume of 10 liters using a thermostat to set the desired temperature. By varying the height difference between the upstream reservoir and outflow, various flow rates were achieved. A minimum flow rate of 1 × 10^−6^ m^3^/s was necessary to sustain constant flow.

Prior to the experiments, the fracture network was fully saturated. Air vents at the fracture ends allowed the release of air and secure full water saturation. Once saturation was achieved under a constant water stream, the air vents were closed. By turning valves, the water flow was switched from tank water to warm water heated by the boiler. To capture possible mixing effects of warm and cold water in the pipes towards the inlet, the inflow temperature was measured by a temperature sensor at the inlet.

### Mathematical model

Heat transport in flowing water *w* can be described by the advection-diffusion-equation derived from the conservation of energy

2 $${\rho }_{w}{C}_{p,w}\frac{\partial {T}_{w}}{\partial t}=-\nabla \left(\,{{\mbox{v}}}\,{\rho }_{w}{C}_{p,w}T\right)+\nabla {\lambda }_{w}\nabla T+{Q}_{w},$$

with water density *ρ*_*w*_, specific heat capacity *C*_*p*,*w*_, temperature *T*_*w*_, time *t*, flow velocity *v*, thermal conductivity *λ*_*w*_, and possible heat sources or sinks *Q*_*w*_.

As we neglect possible turbulence inside the fracture fluid and possible vertical gradients, the heat equation inside an individual fracture can be simplified to one spatial dimension. Heat transport in the solid rock *r* is limited to conduction and therefore the heat equation is given as

3 $${\rho }_{r}{C}_{p,r}\frac{\partial {T}_{r}}{\partial t}=\nabla {\lambda }_{r}\nabla T+{Q}_{r},$$

and is simplified in the current setup to two spatial dimensions.

Both phases do not experience any heat-generating mechanism. Heat transfer between phases is the only sink/source for each phase. Following equation (1), the temperature difference between the phases Δ*T* can be defined as Δ*T* = *T*_*w*_ − *T*_*r*_. Therefore, *Q*_*w*_ = − *Q* and *Q*_*r*_ = *Q*.

An empirical function of the heat transfer coefficient was fitted to the results of over 240 experiments^4,16,20^ and the analysis of those experiments^10^ using the mathematical expression

4 $$h=a\cdot ({(d+{{{{{\rm{exp}}}}}}(-b\cdot \alpha ))}^{-1}\cdot {(e+{{{{{\rm{exp}}}}}}(-c\cdot v))}^{-1})+f\cdot v,$$

with parameters a - f determined using the non-least square fitting algorithm of the Matlab Curve Fitting Toolbox, and fluid flow velocity *v* (m/s) and fracture aperture *α* (m). The best fit has been achieved with the values $a=0.00905\,{{{{{{{\rm{W}}}}}}}}/({{{{{{{{\rm{m}}}}}}}}{}^{2}}^{\circ }{{{{{{{\rm{C}}}}}}}})$, *b* = 2.314 × 10^5^/m, *c* = 136.9 s/m, *d* = 0.0009217, *e* = 0.0693, *f* = 650.0 J/(m${{}^{3}}^{\circ }$C).

### Numerical implementation

Equation (3) is numerically solved using a finite difference approximation using a forward in time - centered in space scheme with an explicit time stepping. Equation (2) is numerically solved using a 1D finite difference scheme with a forward in time - centered in space scheme for the diffusion and an upwind scheme of first order for the advective part. The heat transfer between both phases is calculated for each time step based on the local difference between phase temperatures at the respective time step. The spatial resolution of the simulation results presented here are of 1 mm and 5 m, respectively, for laboratory and field scale in each spatial dimension but the results have been found to be principally independent of the numerical resolution within reasonable bounds. The explicit time step was set to 0.1 s and 10 s respectively, guaranteeing a stable simulation. The fractures are implemented as 1D lines and cells of the numerical grid representing the rock that contains one or more fractures interacting with the respective water temperature by equation (1). The heat transfer area is calculated as the respective area of the fracture within the cell and divided by the volume of the numerical cell. The parameters used in the numerical simulation for the experimental reproduction are given in Supplementary Table 2.

## Supplementary information

Supplementary Information

## Data Availability

The experimental data are provided in the article and in the supplementary material.

## Code availability

The numerical simulations presented here were calculated using the model^55^ deposited at 10.5281/zenodo.7411927.
