# Supplementary material for: Velocity-dependent heat transfer controls temperature in fracture networks
Source: Nat Commun. 2023 Jan 23;14:362. doi: 10.1038/s41467-023-36034-w (PMC9871020; doi:10.1038/s41467-023-36034-w)
Supplement: Supplementary file 1 — Supplementary Information [file 41467_2023_36034_MOESM1_ESM.pdf]

Supplementary Information for the manuscript "Velocity dependent heat transfer controls temperature in fracture networks"

Thomas Heinze[1,*] and Nicola Pastore[2]

[1] *Department of Hydrogeochemistry and Hydrogeology; Institute of Geology, Mineralogy and Geophysics; Ruhr-University Bochum; Universitaetsstr. 150, 44801 Bochum, Germany*

[2] *DICATECh Department of Civil, Environmental, Building Engineering, and Chemistry; Politecnico di Bari; Via Edoardo Orabona 4, 70125 Bari, Italy*

[*] *Corresponding author: Thomas Heinze, thomas.heinze@rub.de*

Supplementary Note 1: Bench-scale experiments

Supplementary Table 1 | Experimental settings Hydraulic flow rates and effective hydraulic apertures from the bench scale experiments. Hydraulic aperture of 0.0037 m. Values agree with previous studies on solute transport on the same fracture network (Cherubini et al., 2012; Cherubini et al. 2014).

Q_0	Q_1	Q_2
$1.835 \times 10^{-6}\,\mathrm{m^3\,s^{-1}}$	$1.404 \times 10^{-6}\,\mathrm{m^3\,s^{-1}}$	$0.431 \times 10^{-6}\,\mathrm{m^3\,s^{-1}}$
$2.325 \times 10^{-6}\,\mathrm{m^3\,s^{-1}}$	$1.768 \times 10^{-6}\,\mathrm{m^3\,s^{-1}}$	$0.557 \times 10^{-6}\,\mathrm{m^3\,s^{-1}}$
$2.462 \times 10^{-6}\,\mathrm{m^3\,s^{-1}}$	$1.869 \times 10^{-6}\,\mathrm{m^3\,s^{-1}}$	$0.593 \times 10^{-6}\,\mathrm{m^3\,s^{-1}}$
$2.605 \times 10^{-6}\,\mathrm{m^3\,s^{-1}}$	$1.974 \times 10^{-6}\,\mathrm{m^3\,s^{-1}}$	$0.630 \times 10^{-6}\,\mathrm{m^3\,s^{-1}}$
$2.680 \times 10^{-6}\,\mathrm{m^3\,s^{-1}}$	$2.029 \times 10^{-6}\,\mathrm{m^3\,s^{-1}}$	$0.650 \times 10^{-6}\,\mathrm{m^3\,s^{-1}}$
$2.800 \times 10^{-6}\,\mathrm{m^3\,s^{-1}}$	$2.118 \times 10^{-6}\,\mathrm{m^3\,s^{-1}}$	$0.683 \times 10^{-6}\,\mathrm{m^3\,s^{-1}}$
$2.847 \times 10^{-6}\,\mathrm{m^3\,s^{-1}}$	$2.152 \times 10^{-6}\,\mathrm{m^3\,s^{-1}}$	$0.695 \times 10^{-6}\,\mathrm{m^3\,s^{-1}}$
$3.002 \times 10^{-6}\,\mathrm{m^3\,s^{-1}}$	$2.265 \times 10^{-6}\,\mathrm{m^3\,s^{-1}}$	$0.737 \times 10^{-6}\,\mathrm{m^3\,s^{-1}}$
$3.998 \times 10^{-6}\,\mathrm{m^3\,s^{-1}}$	$2.985 \times 10^{-6}\,\mathrm{m^3\,s^{-1}}$	$1.013 \times 10^{-6}\,\mathrm{m^3\,s^{-1}}$
$4.030 \times 10^{-6}\,\mathrm{m^3\,s^{-1}}$	$3.008 \times 10^{-6}\,\mathrm{m^3\,s^{-1}}$	$1.022 \times 10^{-6}\,\mathrm{m^3\,s^{-1}}$
$4.217 \times 10^{-6}\,\mathrm{m^3\,s^{-1}}$	$3.142 \times 10^{-6}\,\mathrm{m^3\,s^{-1}}$	$1.075 \times 10^{-6}\,\mathrm{m^3\,s^{-1}}$
$4.225 \times 10^{-6}\,\mathrm{m^3\,s^{-1}}$	$3.148 \times 10^{-6}\,\mathrm{m^3\,s^{-1}}$	$1.078 \times 10^{-6}\,\mathrm{m^3\,s^{-1}}$
$4.471 \times 10^{-6}\,\mathrm{m^3\,s^{-1}}$	$3.323 \times 10^{-6}\,\mathrm{m^3\,s^{-1}}$	$1.148 \times 10^{-6}\,\mathrm{m^3\,s^{-1}}$
$5.837 \times 10^{-6}\,\mathrm{m^3\,s^{-1}}$	$4.289 \times 10^{-6}\,\mathrm{m^3\,s^{-1}}$	$1.548 \times 10^{-6}\,\mathrm{m^3\,s^{-1}}$
$5.880 \times 10^{-6}\,\mathrm{m^3\,s^{-1}}$	$4.319 \times 10^{-6}\,\mathrm{m^3\,s^{-1}}$	$1.561 \times 10^{-6}\,\mathrm{m^3\,s^{-1}}$
$6.445 \times 10^{-6}\,\mathrm{m^3\,s^{-1}}$	$4.715 \times 10^{-6}\,\mathrm{m^3\,s^{-1}}$	$1.730 \times 10^{-6}\,\mathrm{m^3\,s^{-1}}$
$7.056 \times 10^{-6}\,\mathrm{m^3\,s^{-1}}$	$5.141 \times 10^{-6}\,\mathrm{m^3\,s^{-1}}$	$1.916 \times 10^{-6}\,\mathrm{m^3\,s^{-1}}$
$7.959 \times 10^{-6}\,\mathrm{m^3\,s^{-1}}$	$5.767 \times 10^{-6}\,\mathrm{m^3\,s^{-1}}$	$2.192 \times 10^{-6}\,\mathrm{m^3\,s^{-1}}$
$8.971 \times 10^{-6}\,\mathrm{m^3\,s^{-1}}$	$6.465 \times 10^{-6}\,\mathrm{m^3\,s^{-1}}$	$2.506 \times 10^{-6}\,\mathrm{m^3\,s^{-1}}$
$1.236 \times 10^{-5}\,\mathrm{m^3\,s^{-1}}$	$0.877 \times 10^{-5}\,\mathrm{m^3\,s^{-1}}$	$0.359 \times 10^{-5}\,\mathrm{m^3\,s^{-1}}$
$1.259 \times 10^{-5}\,\mathrm{m^3\,s^{-1}}$	$0.893 \times 10^{-5}\,\mathrm{m^3\,s^{-1}}$	$0.367 \times 10^{-5}\,\mathrm{m^3\,s^{-1}}$

Supplementary Table 2 | Physical parameters Values used in the numerical simulations.

Physical quantity	Symbol	Value
Thermal conductivity of rock	λ_r	$3.0\,\mathrm{W\,m^{-1}\,°C^{-1}}$
Thermal conductivity of water	λ_w	$0.6\,\mathrm{W\,m^{-1}\,°C^{-1}}$
Specific heat capacity of rock	$C_{p,r}$	$910\,\mathrm{J\,kg^{-1}\,°C^{-1}}$
Specific heat capacity of water	$C_{p.w}$	$4200\,\mathrm{J\,kg^{-1}\,°C^{-1}}$
Density of rock	ρ_r	$2500\,\mathrm{kg\,m^{-3}}$
Density of water	ρ_w	$1000\,\mathrm{kg\,m^{-3}}$

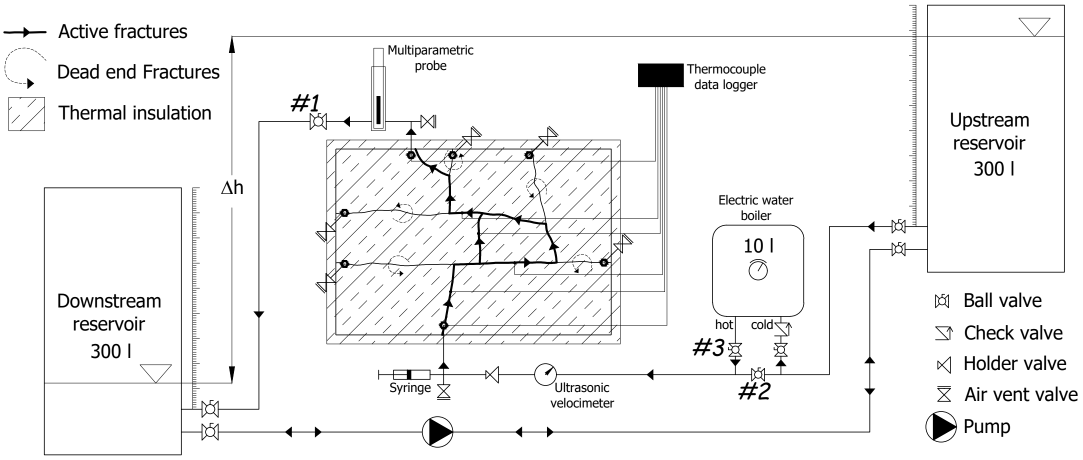

Supplementary Figure 1: Schematic sketch of the experimental setup with all used equipment.

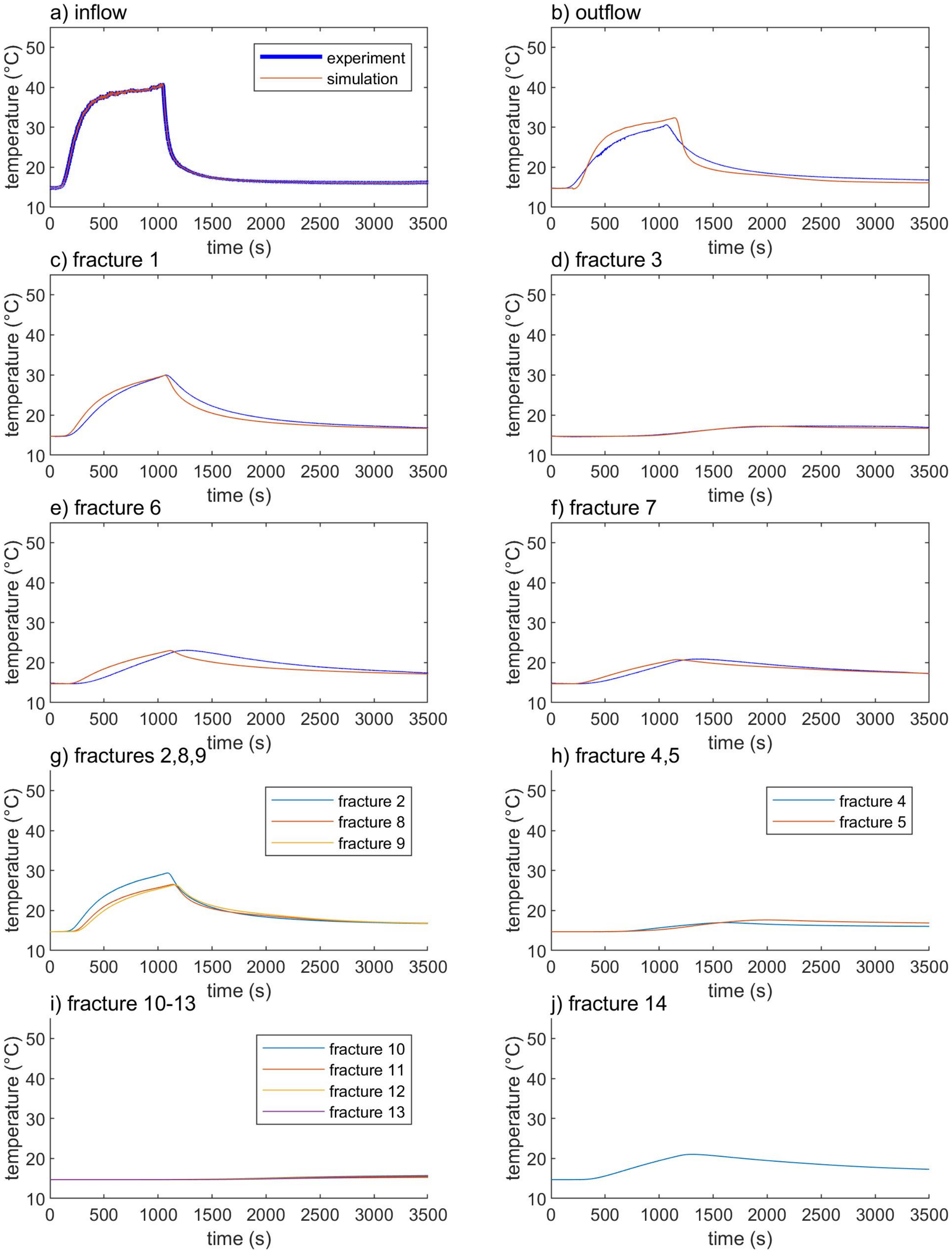

Supplementary Figure 2: Temperatures recorded at six locations in the setup for a flow rate of $1.835 \times 10^{-6}\ \mathrm{m^3\,s^{-1}}$ during the experiment and the numerical simulation at these six locations and in the middle of the remaining fractures.

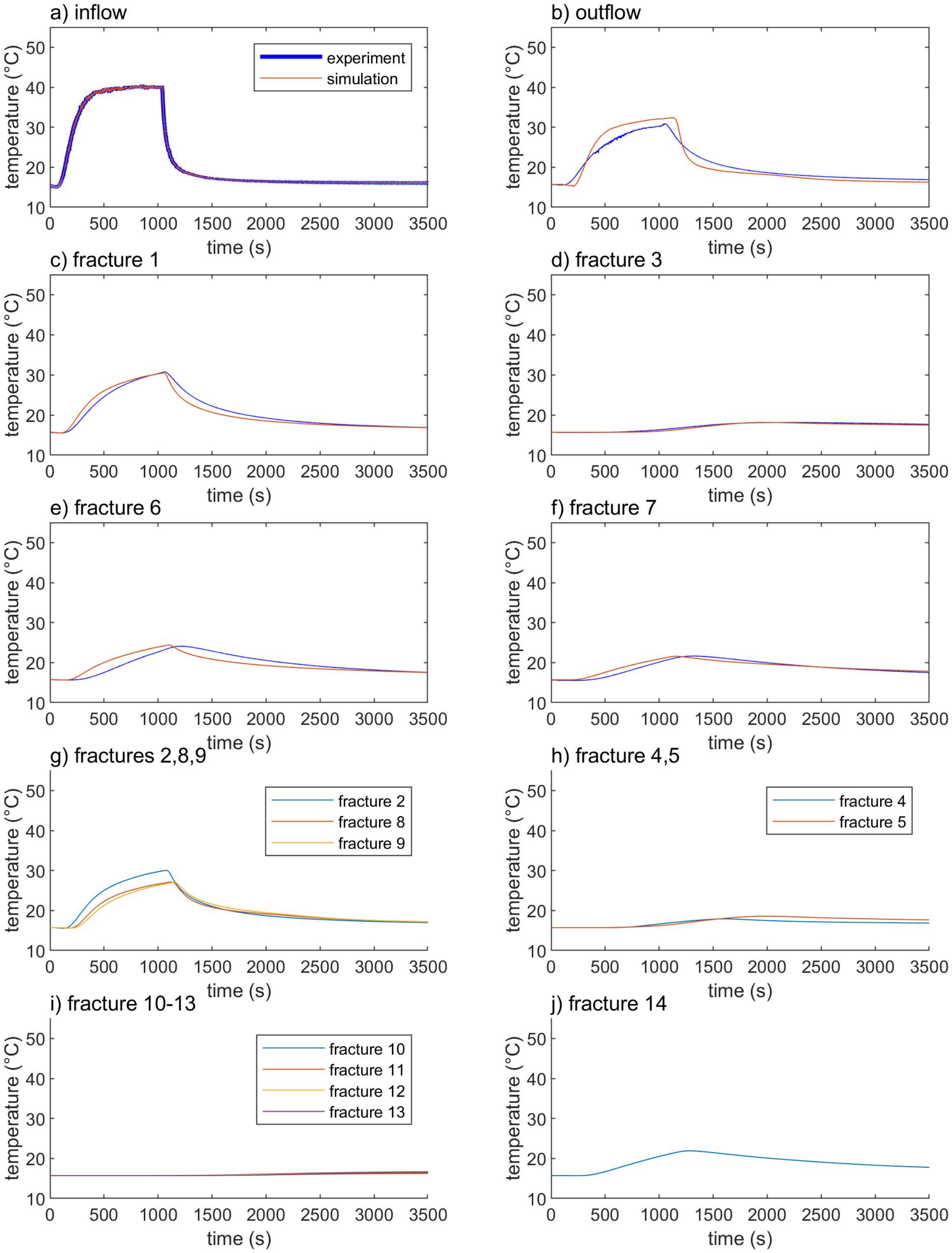

Supplementary Figure 3: Temperatures recorded at six locations in the setup for a flow rate of $2.325 \times 10^{-6}\ \mathrm{m}^3\,\mathrm{s}^{-1}$ during the experiment and the numerical simulation at these six locations and in the middle of the remaining fractures.

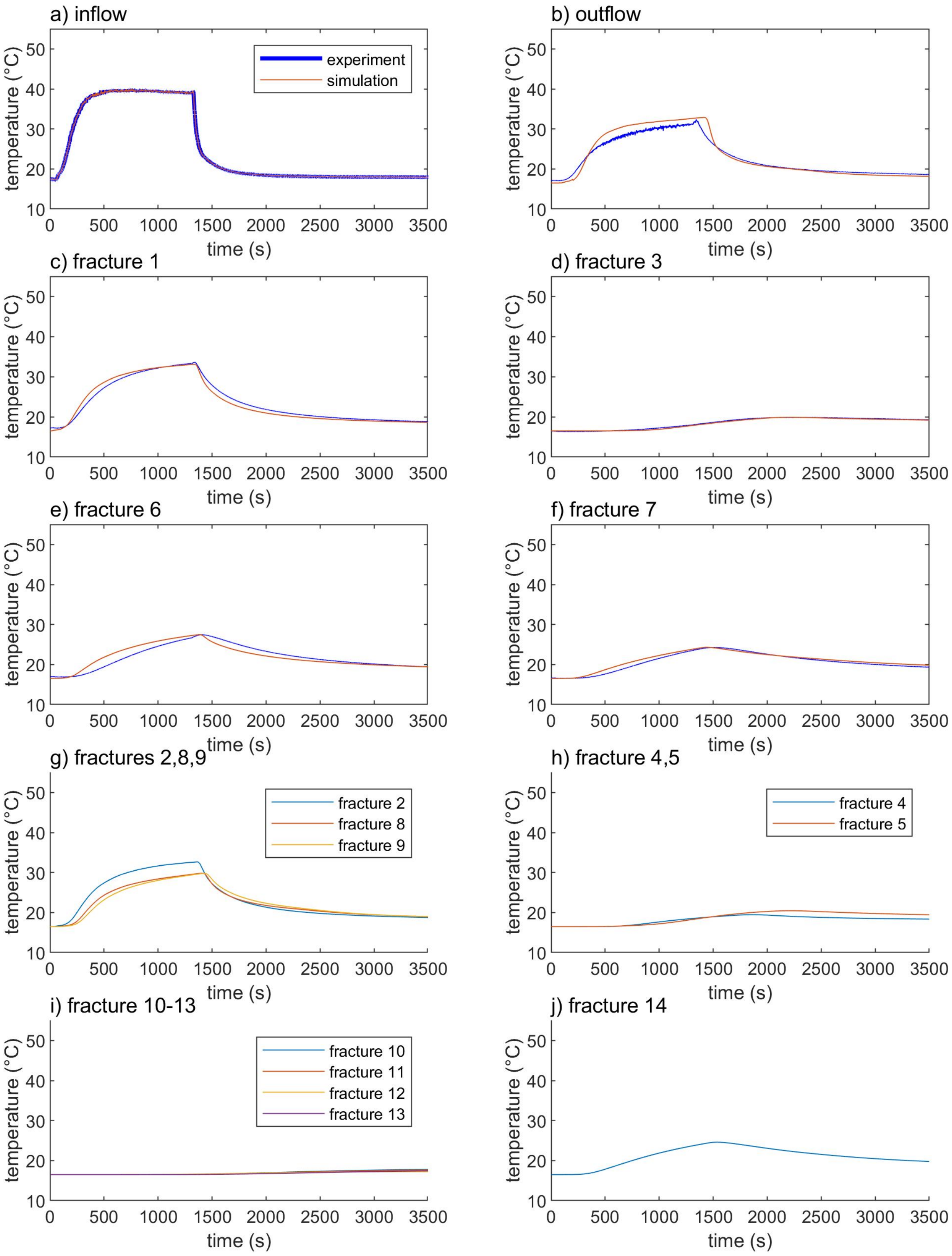

Supplementary Figure 4: Temperatures recorded at six locations in the setup for a flow rate of $2.462 \times 10^{-6}\,\mathrm{m}^3\,\mathrm{s}^{-1}$ during the experiment and the numerical simulation at these six locations and in the middle of the remaining fractures.

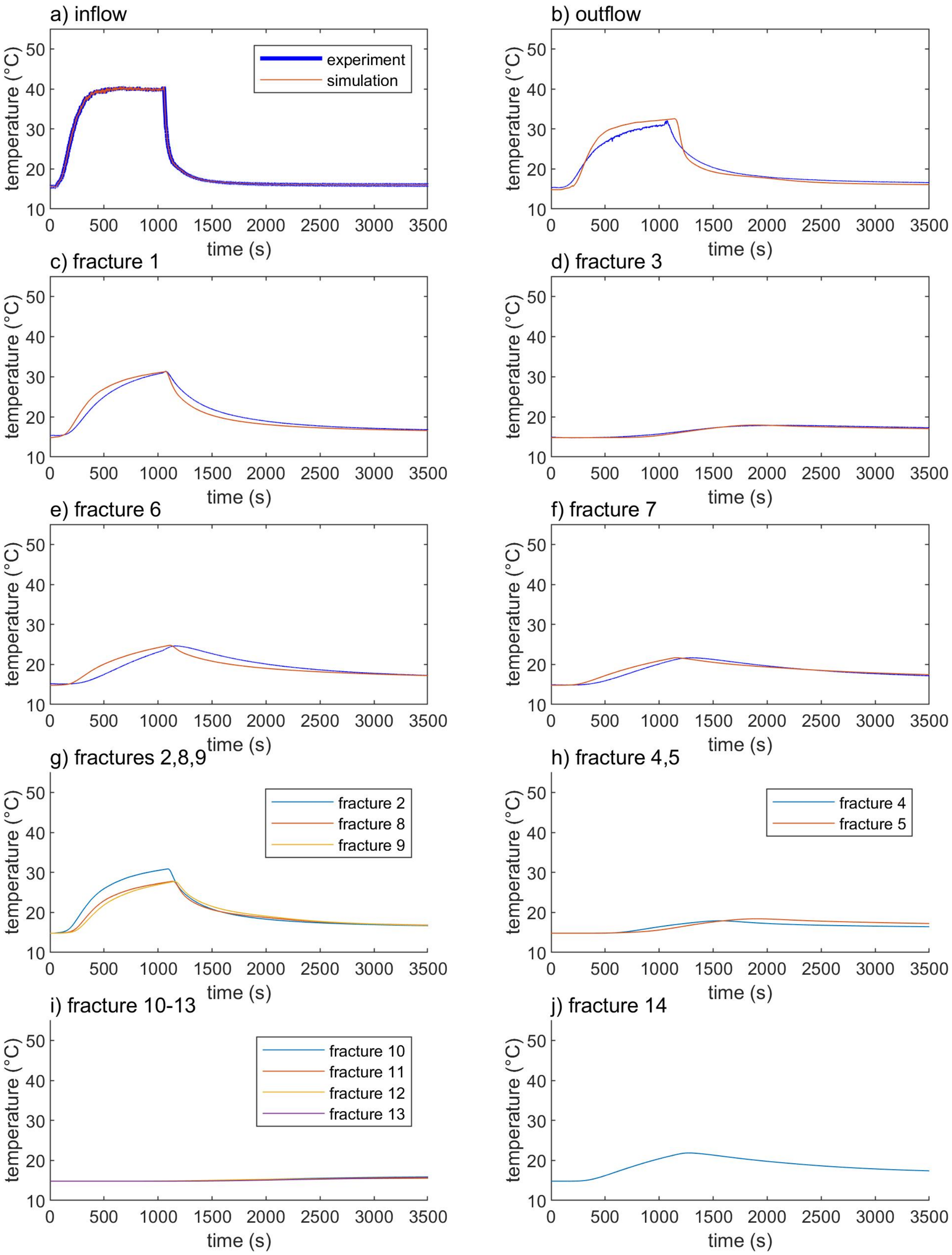

Supplementary Figure 5: Temperatures recorded at six locations in the setup for a flow rate of $2.605 \times 10^{-6}\,\mathrm{m^3\,s^{-1}}$ during the experiment and the numerical simulation at these six locations and in the middle of the remaining fractures.

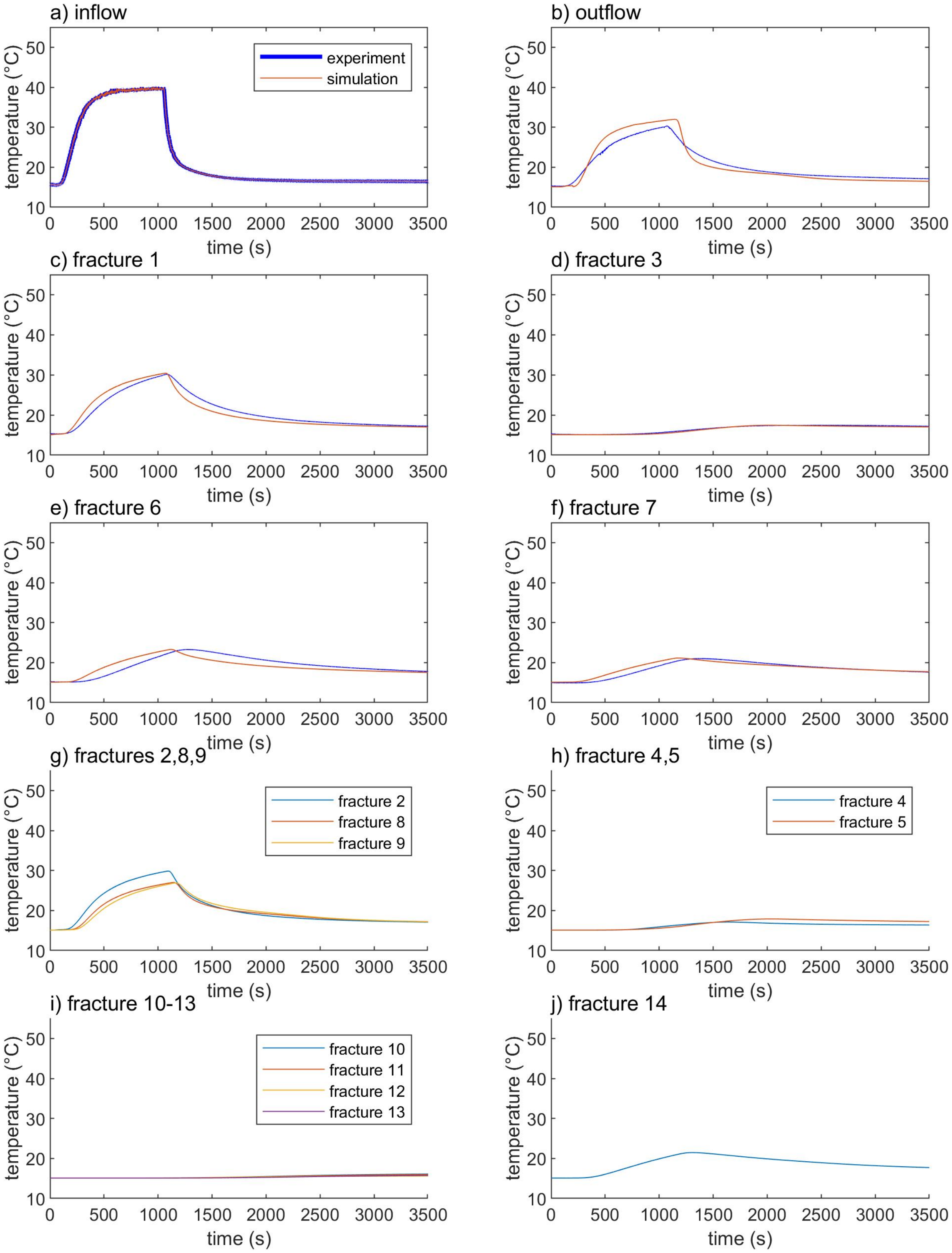

Supplementary Figure 6: Temperatures recorded at six locations in the setup for a flow rate of $2.680 \times 10^{-6}\,\mathrm{m}^3\,\mathrm{s}^{-1}$ during the experiment and the numerical simulation at these six locations and in the middle of the remaining fractures.

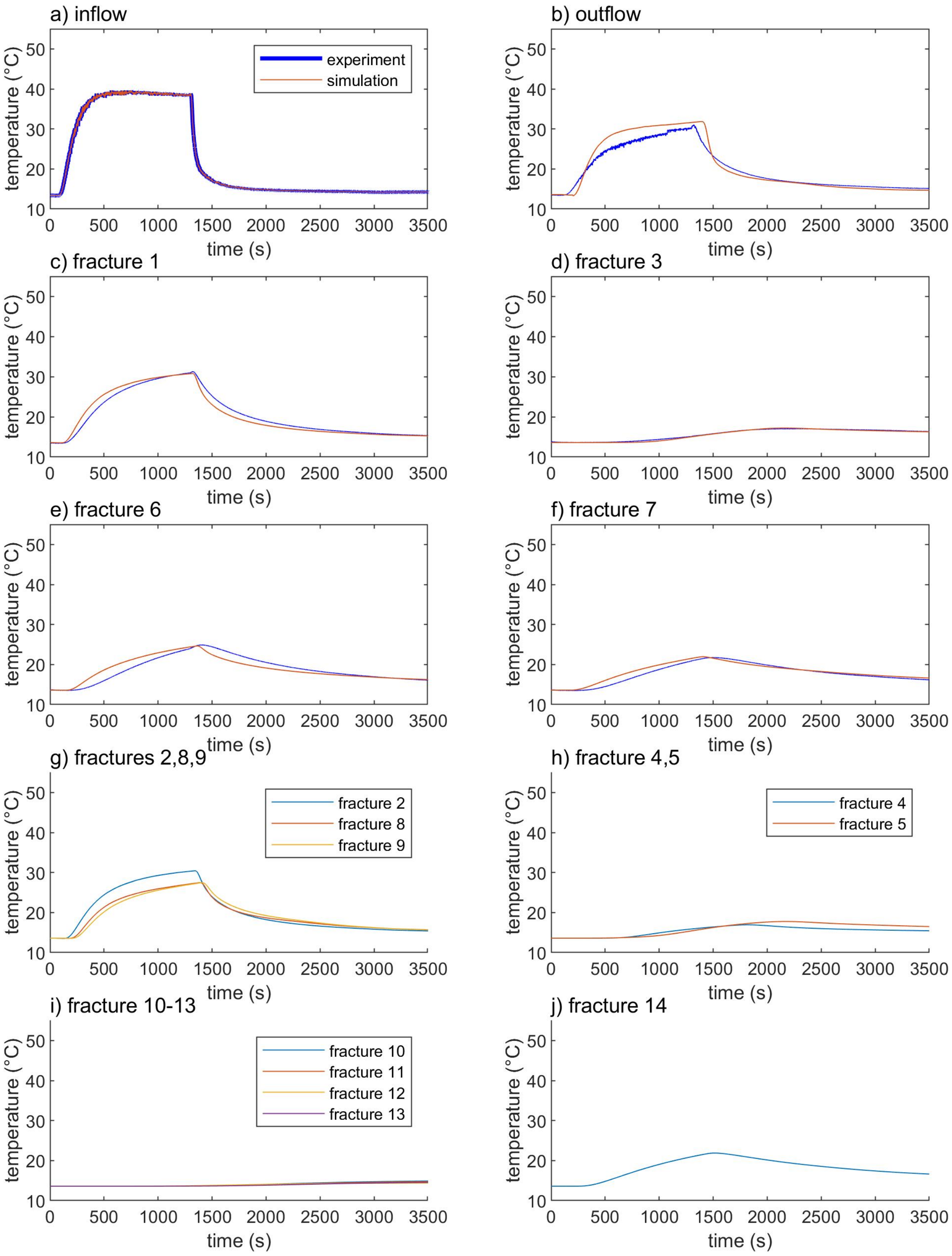

Supplementary Figure 7: Temperatures recorded at six locations in the setup for a flow rate of $2.800 \times 10^{-6}\,\mathrm{m^3\,s^{-1}}$ during the experiment and the numerical simulation at these six locations and in the middle of the remaining fractures.

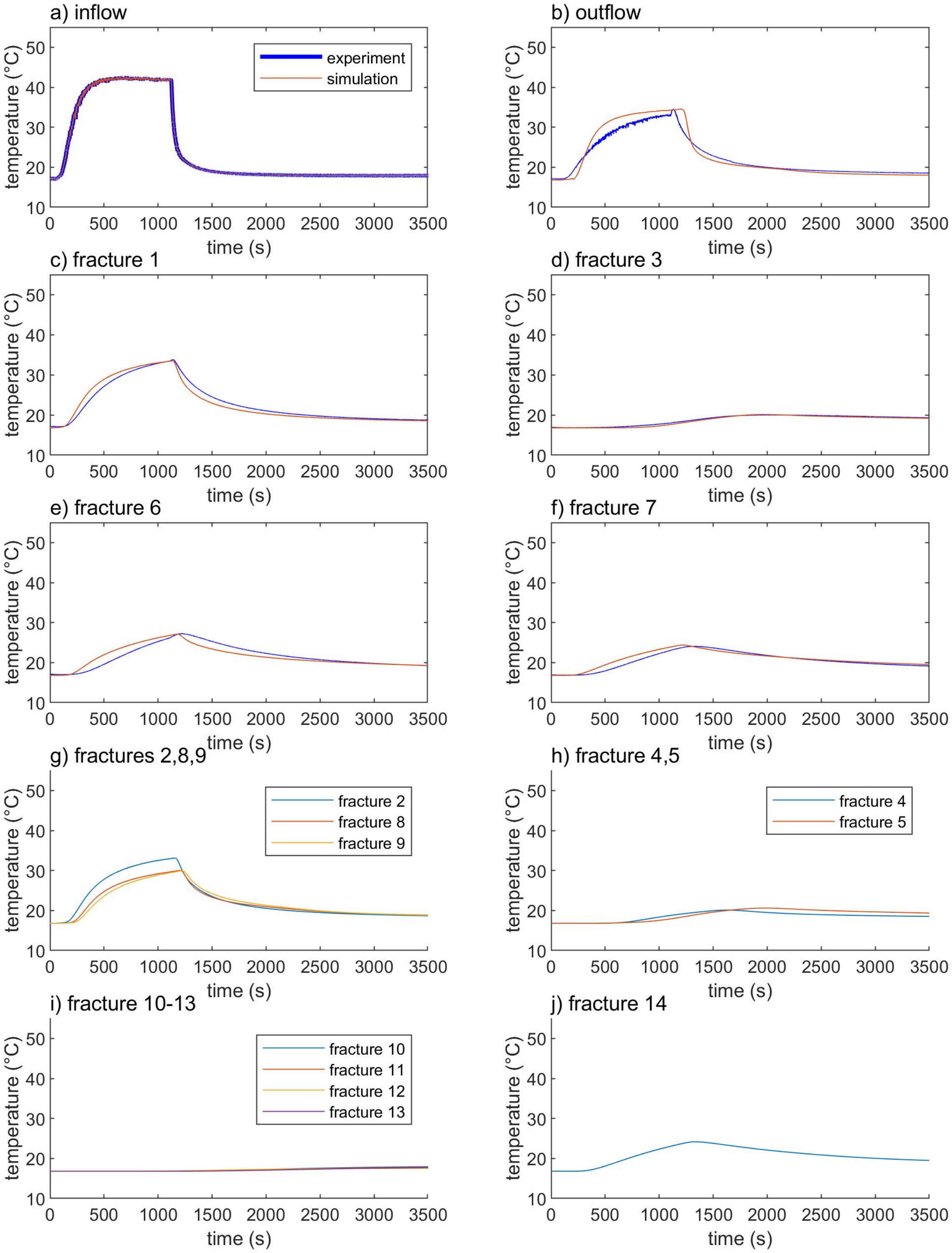

Supplementary Figure 8: Temperatures recorded at six locations in the setup for a flow rate of $2.847 \times 10^{-6}\ \mathrm{m^3\,s^{-1}}$ during the experiment and the numerical simulation at these six locations and in the middle of the remaining fractures.

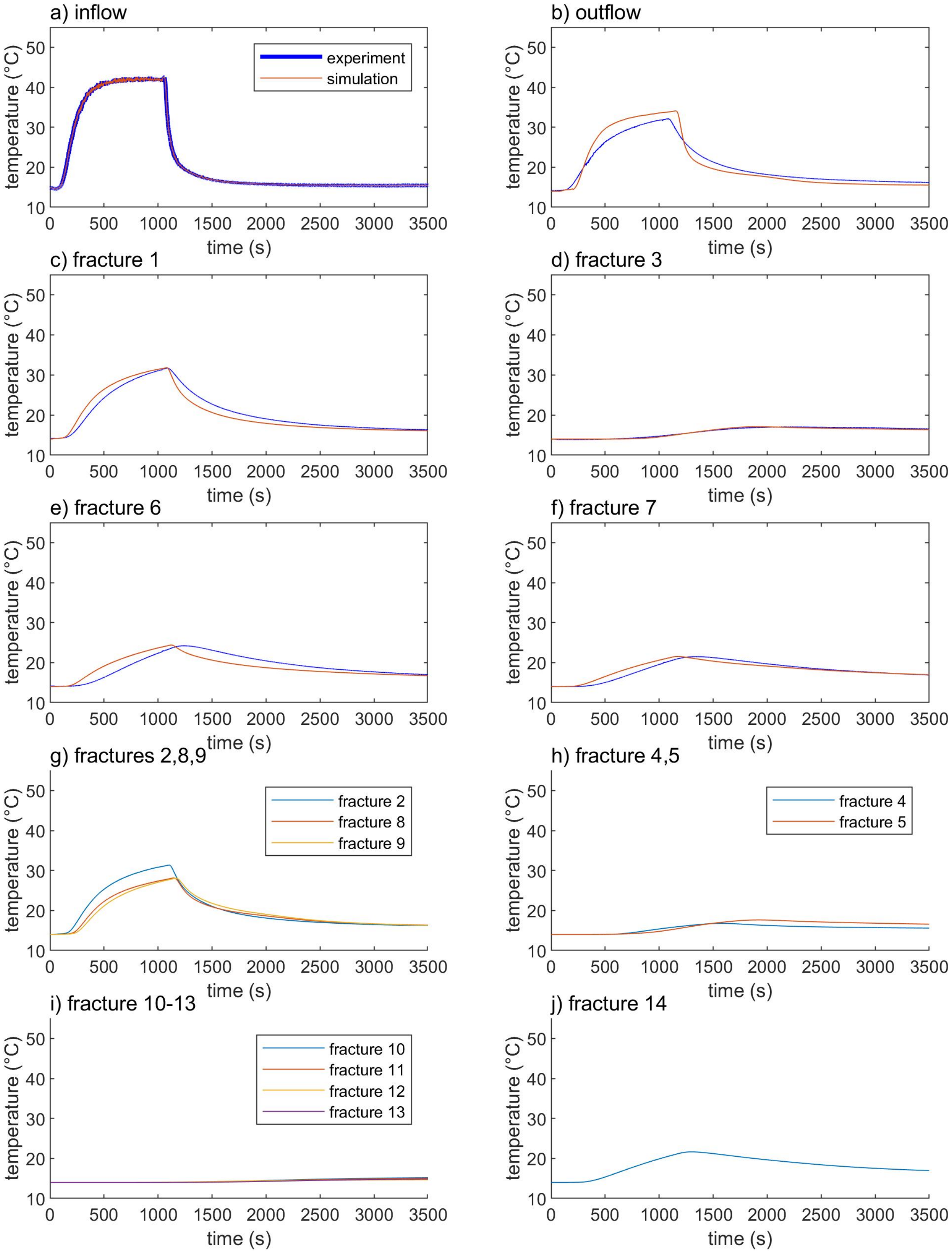

Supplementary Figure 9: Temperatures recorded at six locations in the setup for a flow rate of $3.003 \times 10^{-6}\,\mathrm{m^3\,s^{-1}}$ during the experiment and the numerical simulation at these six locations and in the middle of the remaining fractures.

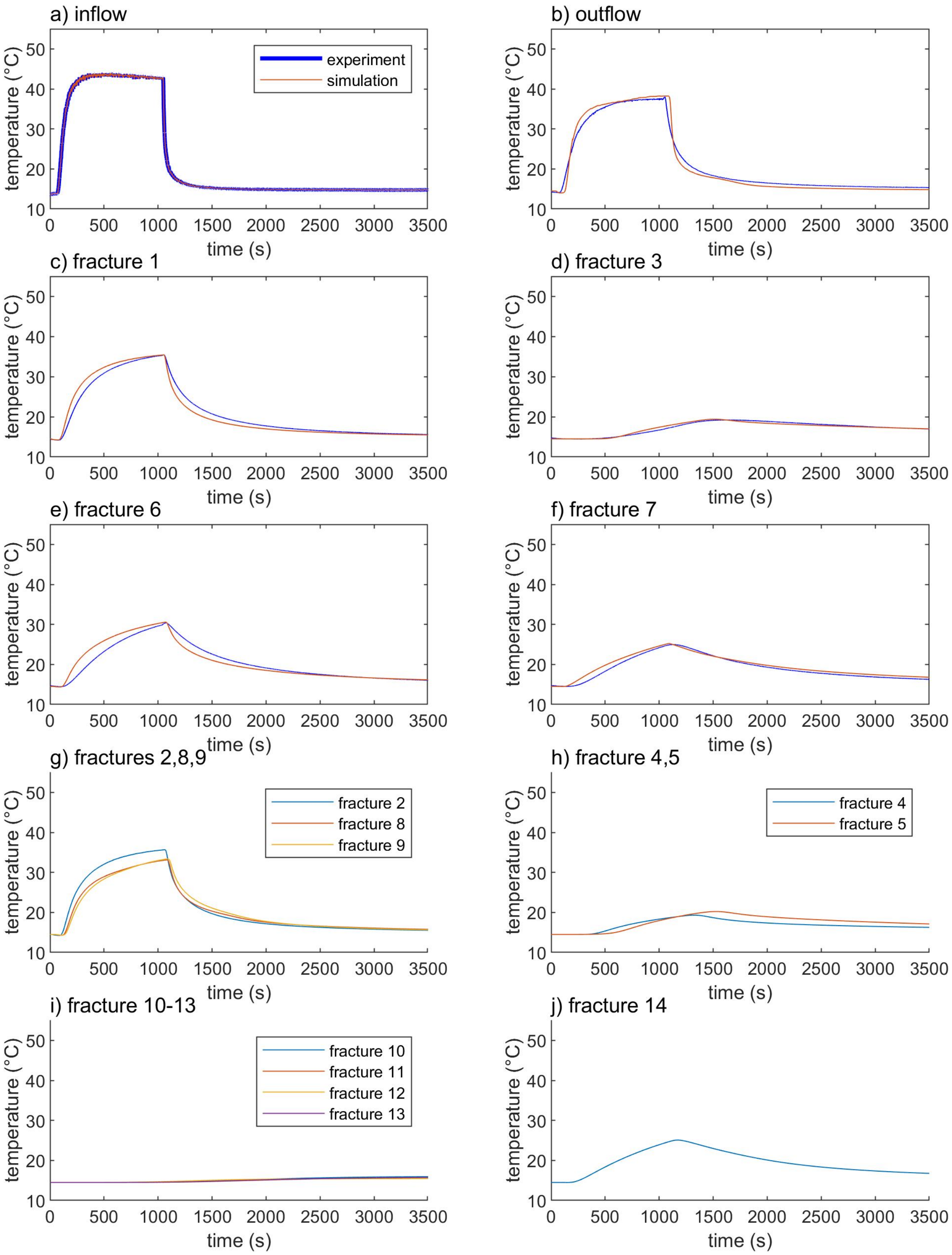

Supplementary Figure 10: Temperatures recorded at six locations in the setup for a flow rate of $3.998 \times 10^{-6}\ \mathrm{m}^3\,\mathrm{s}^{-1}$ during the experiment and the numerical simulation at these six locations and in the middle of the remaining fractures.

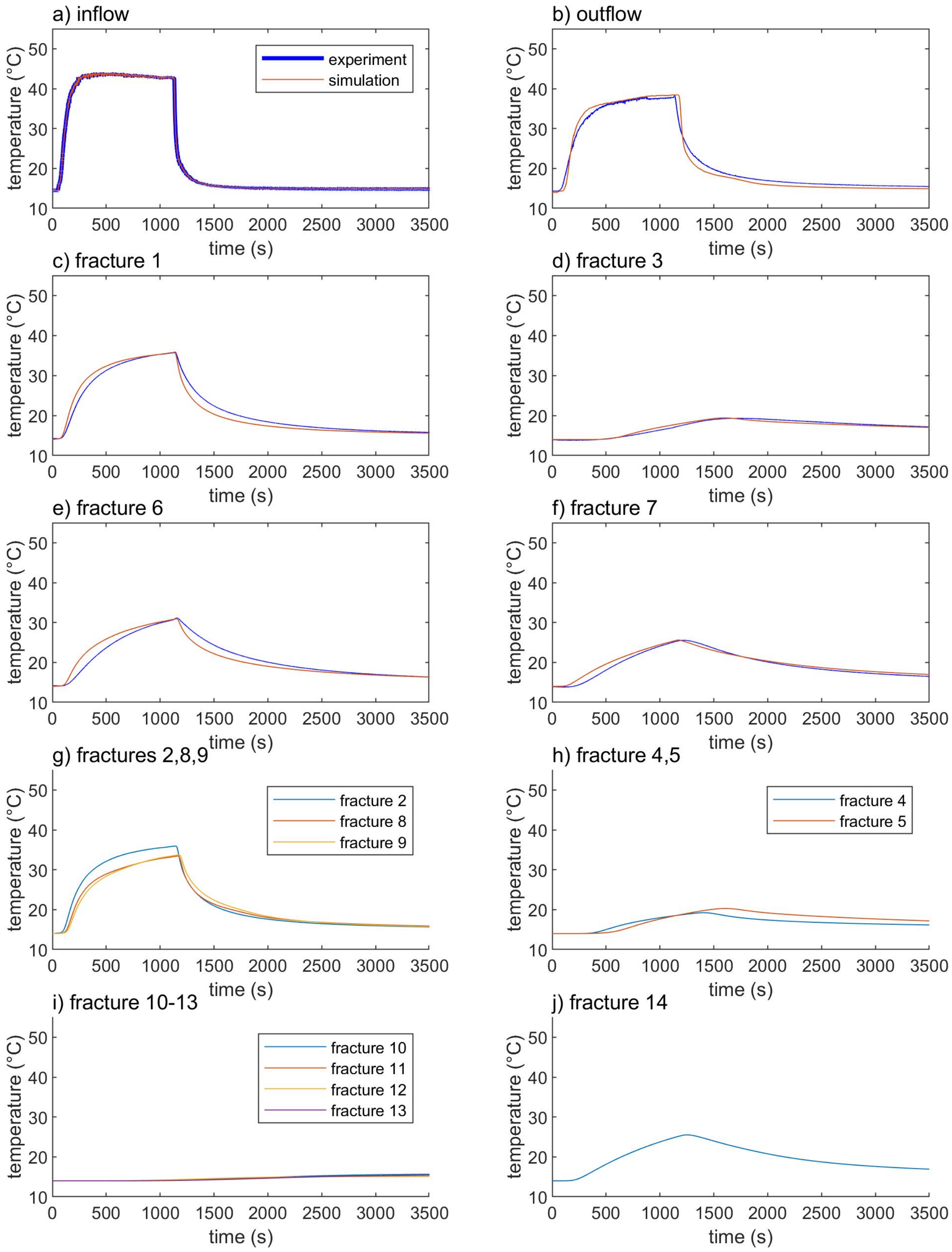

Supplementary Figure 11: Temperatures recorded at six locations in the setup for a flow rate of $4.030 \times 10^{-6}\,\mathrm{m^3\,s^{-1}}$ during the experiment and the numerical simulation at these six locations and in the middle of the remaining fractures.

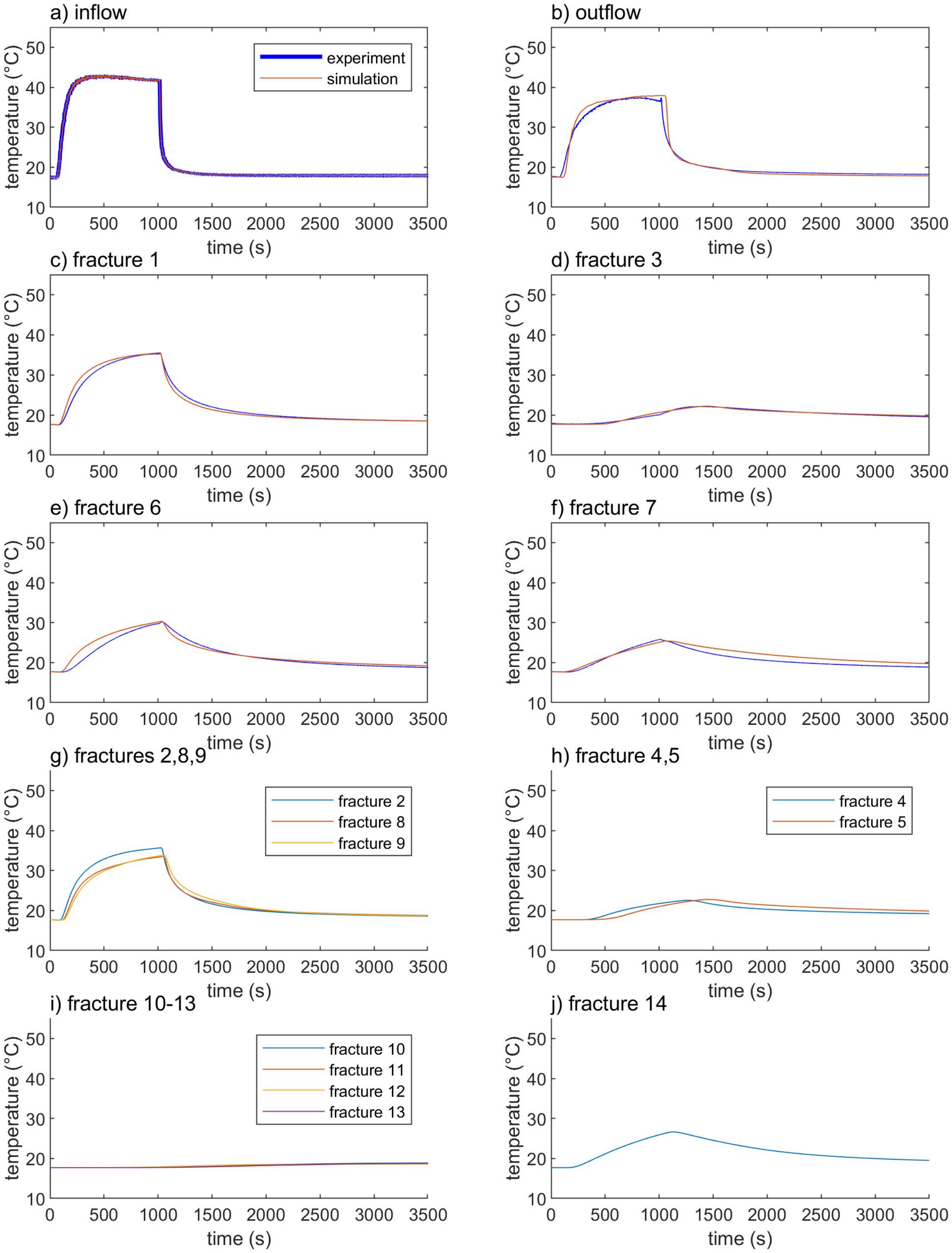

Supplementary Figure 12: Temperatures recorded at six locations in the setup for a flow rate of $4.217 \times 10^{-6}\ \mathrm{m^3\,s^{-1}}$ during the experiment and the numerical simulation at these six locations and in the middle of the remaining fractures.

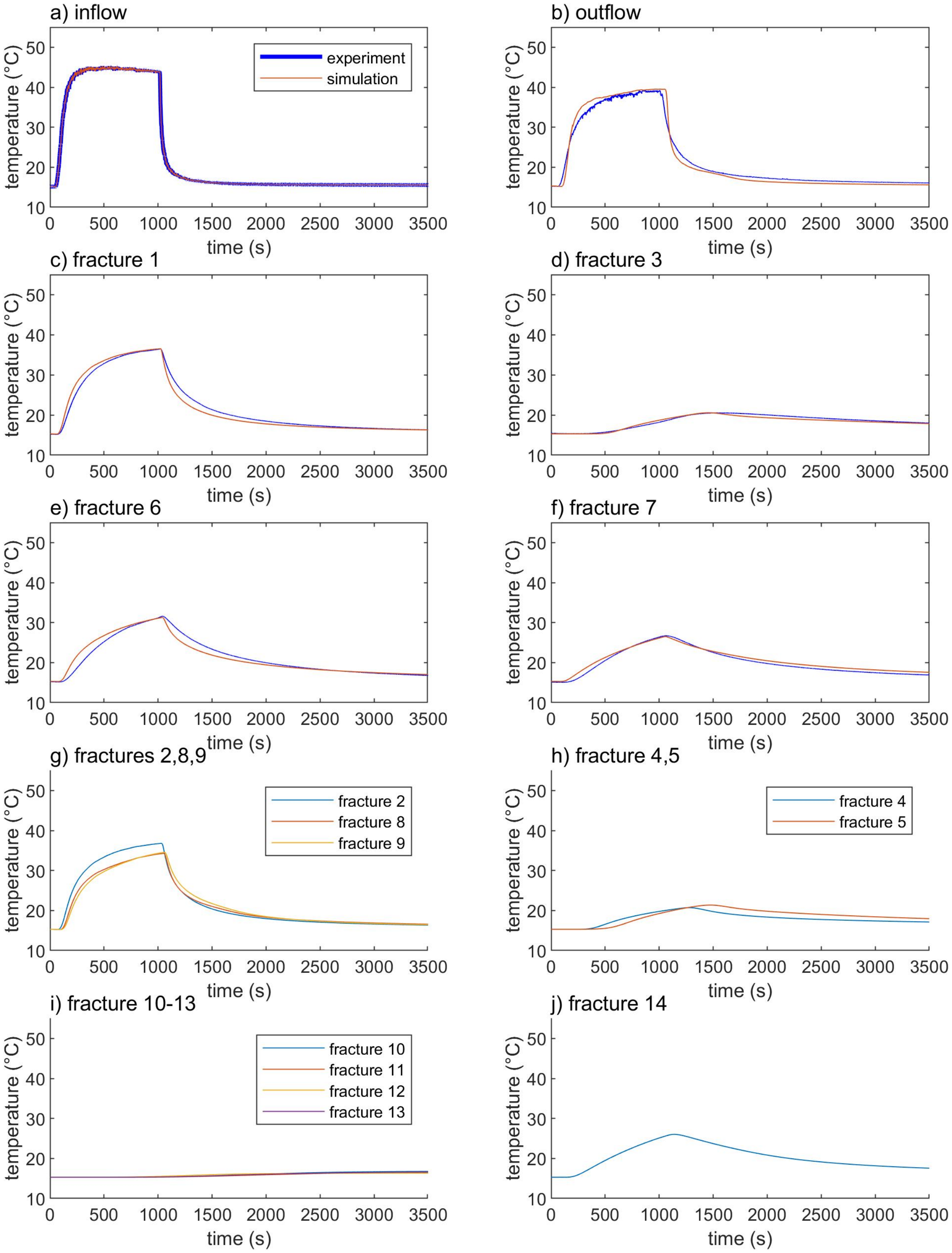

Supplementary Figure 13: Temperatures recorded at six locations in the setup for a flow rate of $4.225 \times 10^{-6}\ \mathrm{m^3\,s^{-1}}$ during the experiment and the numerical simulation at these six locations and in the middle of the remaining fractures.

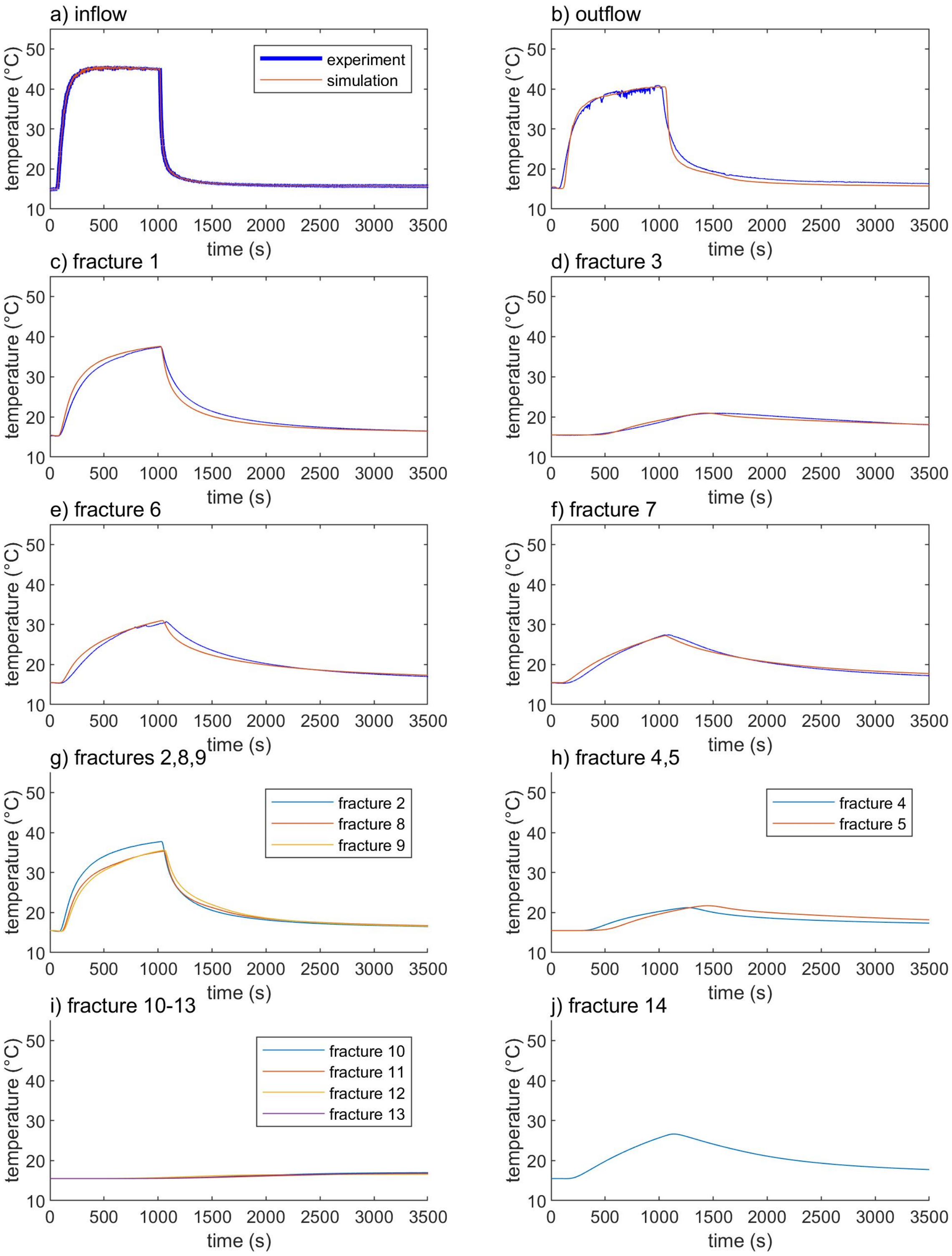

Supplementary Figure 14: Temperatures recorded at six locations in the setup for a flow rate of $4.471 \times 10^{-6}\ \mathrm{m}^3\,\mathrm{s}^{-1}$ during the experiment and the numerical simulation at these six locations and in the middle of the remaining fractures.

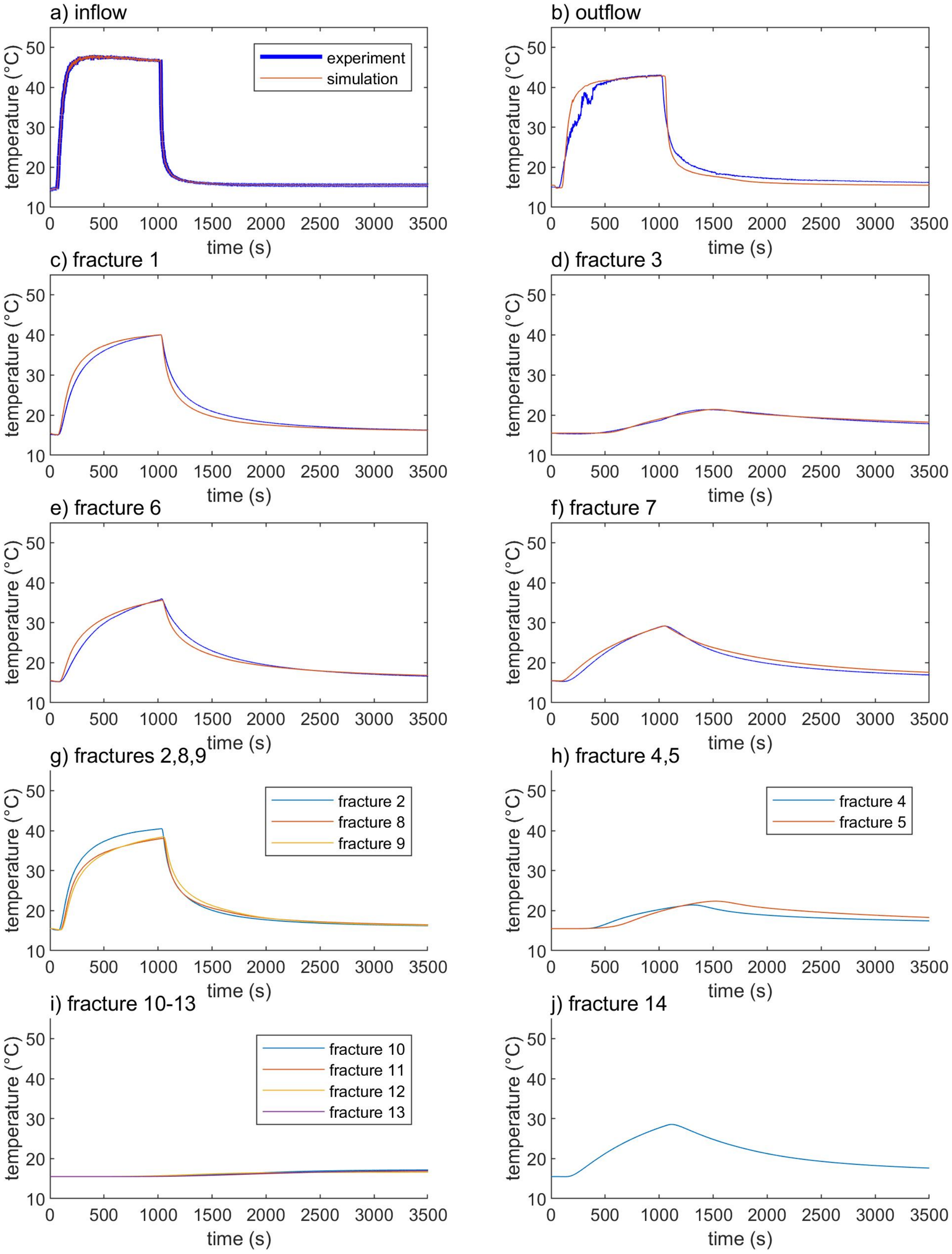

Supplementary Figure 15: Temperatures recorded at six locations in the setup for a flow rate of $5.837 \times 10^{-6}\,\mathrm{m^3\,s^{-1}}$ during the experiment and the numerical simulation at these six locations and in the middle of the remaining fractures.

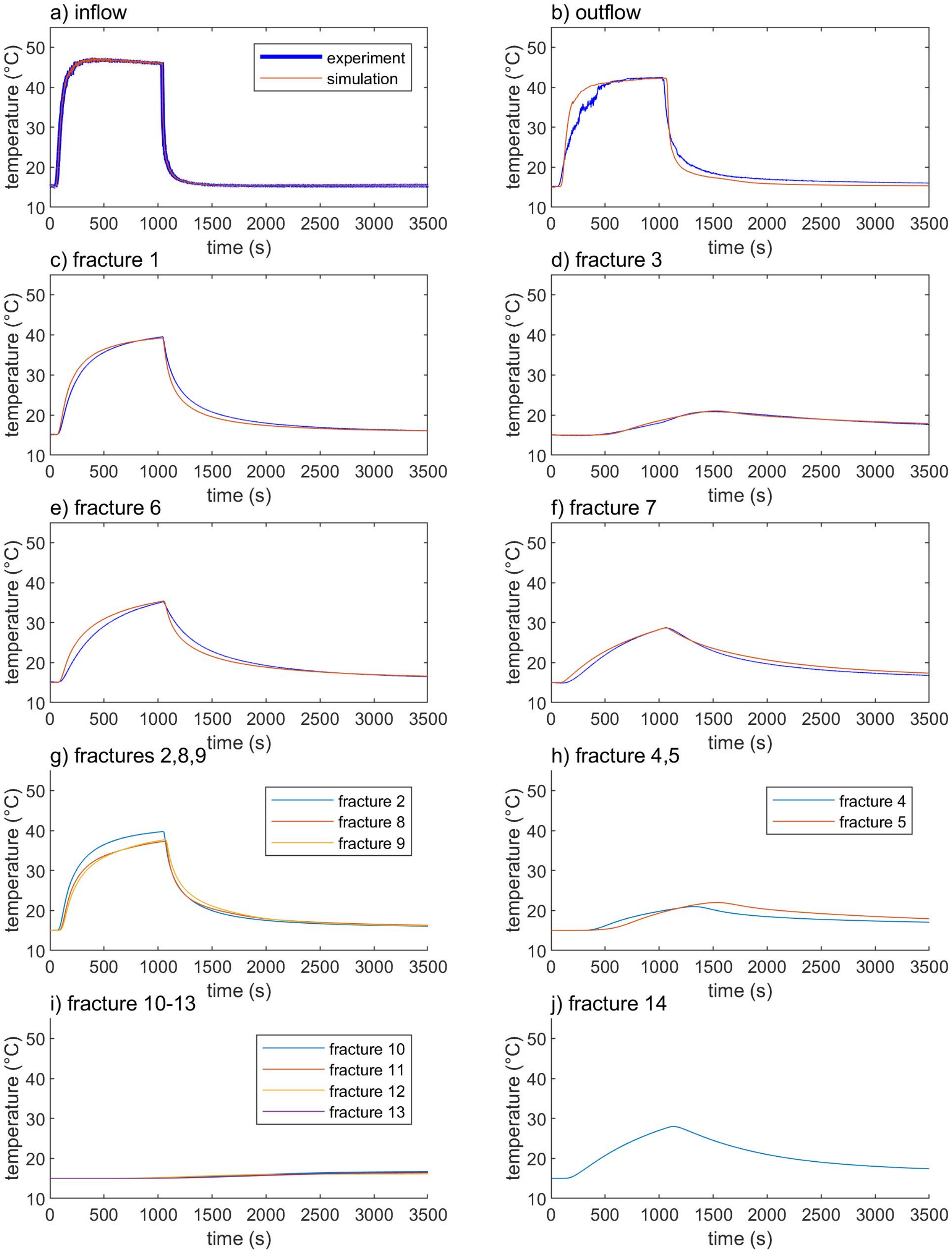

Supplementary Figure 16: Temperatures recorded at six locations in the setup for a flow rate of $5.880 \times 10^{-6}\ \mathrm{m^3\,s^{-1}}$ during the experiment and the numerical simulation at these six locations and in the middle of the remaining fractures.

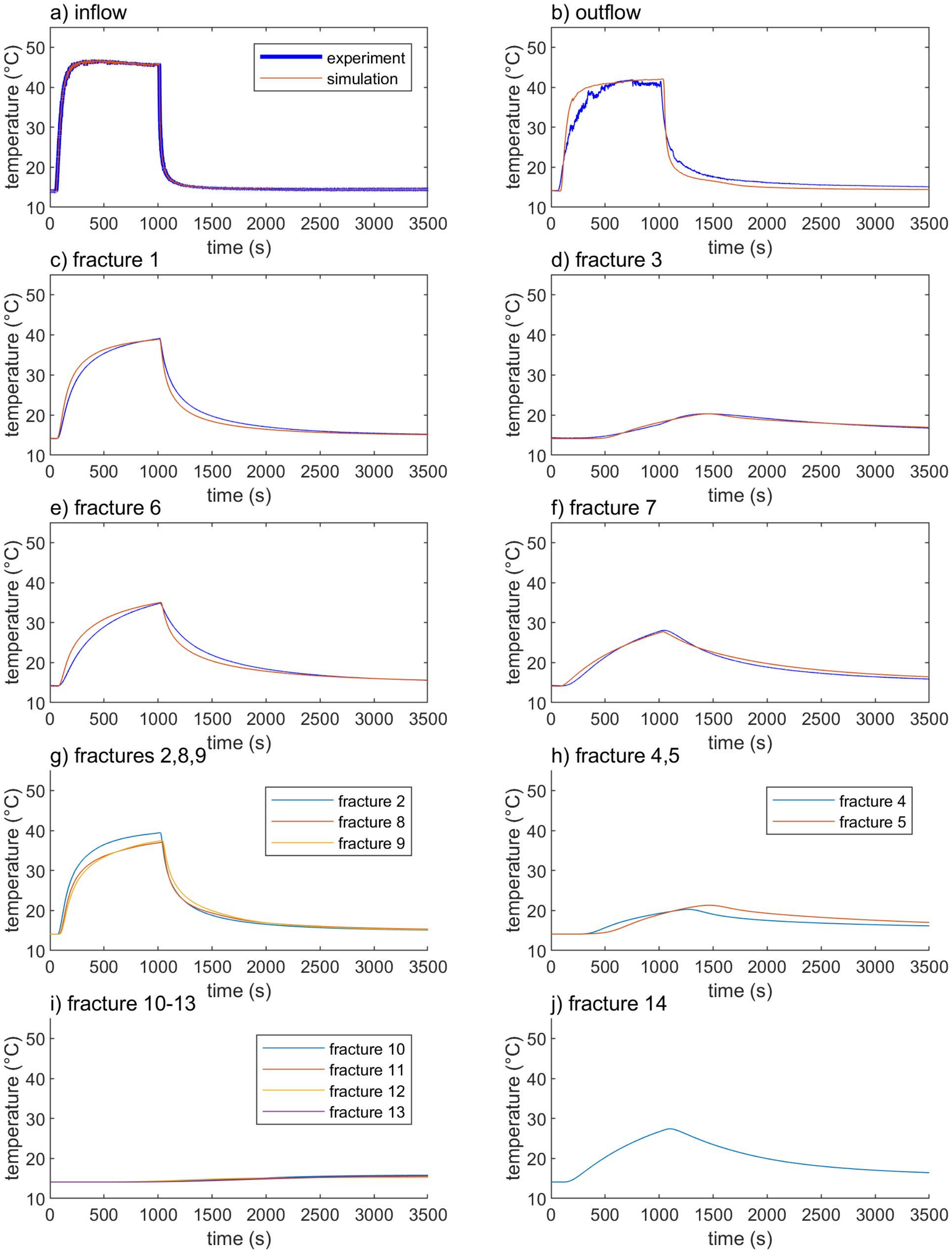

Supplementary Figure 17: Temperatures recorded at six locations in the setup for a flow rate of $6.445 \times 10^{-6}\,\mathrm{m}^3\,\mathrm{s}^{-1}$ during the experiment and the numerical simulation at these six locations and in the middle of the remaining fractures.

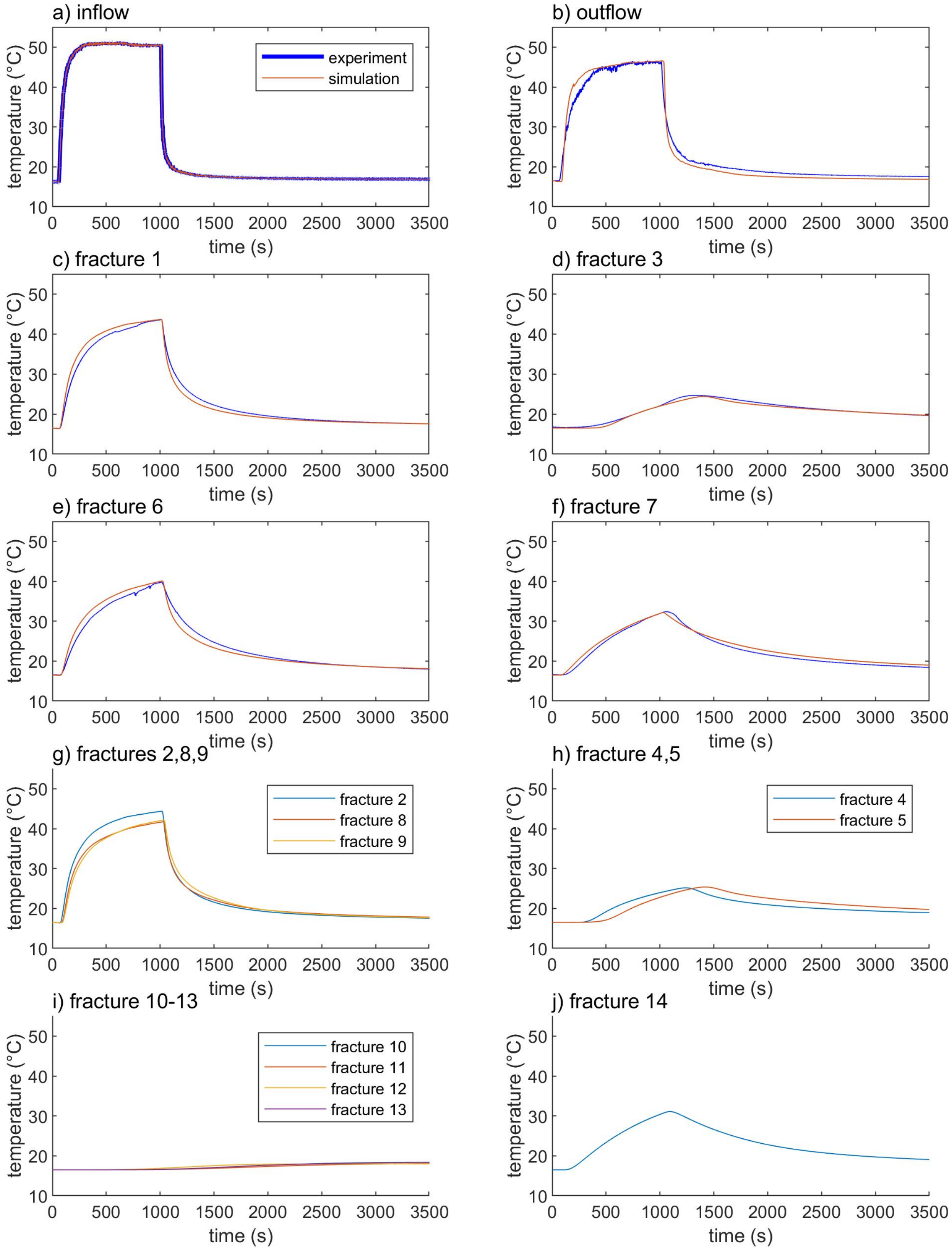

Supplementary Figure 18: Temperatures recorded at six locations in the setup for a flow rate of 7.056×10^{-6} m^3 s^{-1} during the experiment and the numerical simulation at these six locations and in the middle of the remaining fractures.

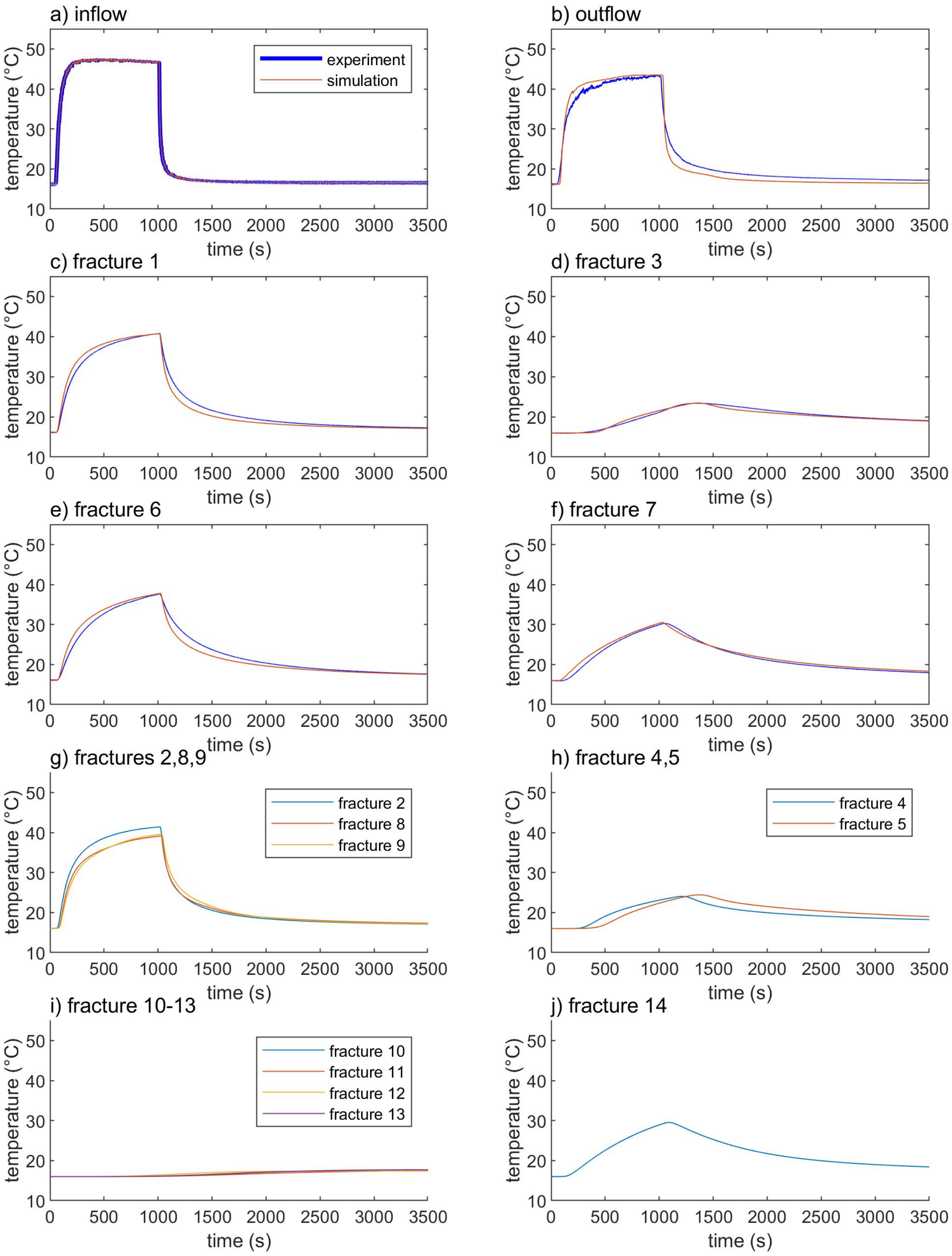

Supplementary Figure 19: Temperatures recorded at six locations in the setup for a flow rate of $7.959 \times 10^{-6}\ \mathrm{m^3\,s^{-1}}$ during the experiment and the numerical simulation at these six locations and in the middle of the remaining fractures.

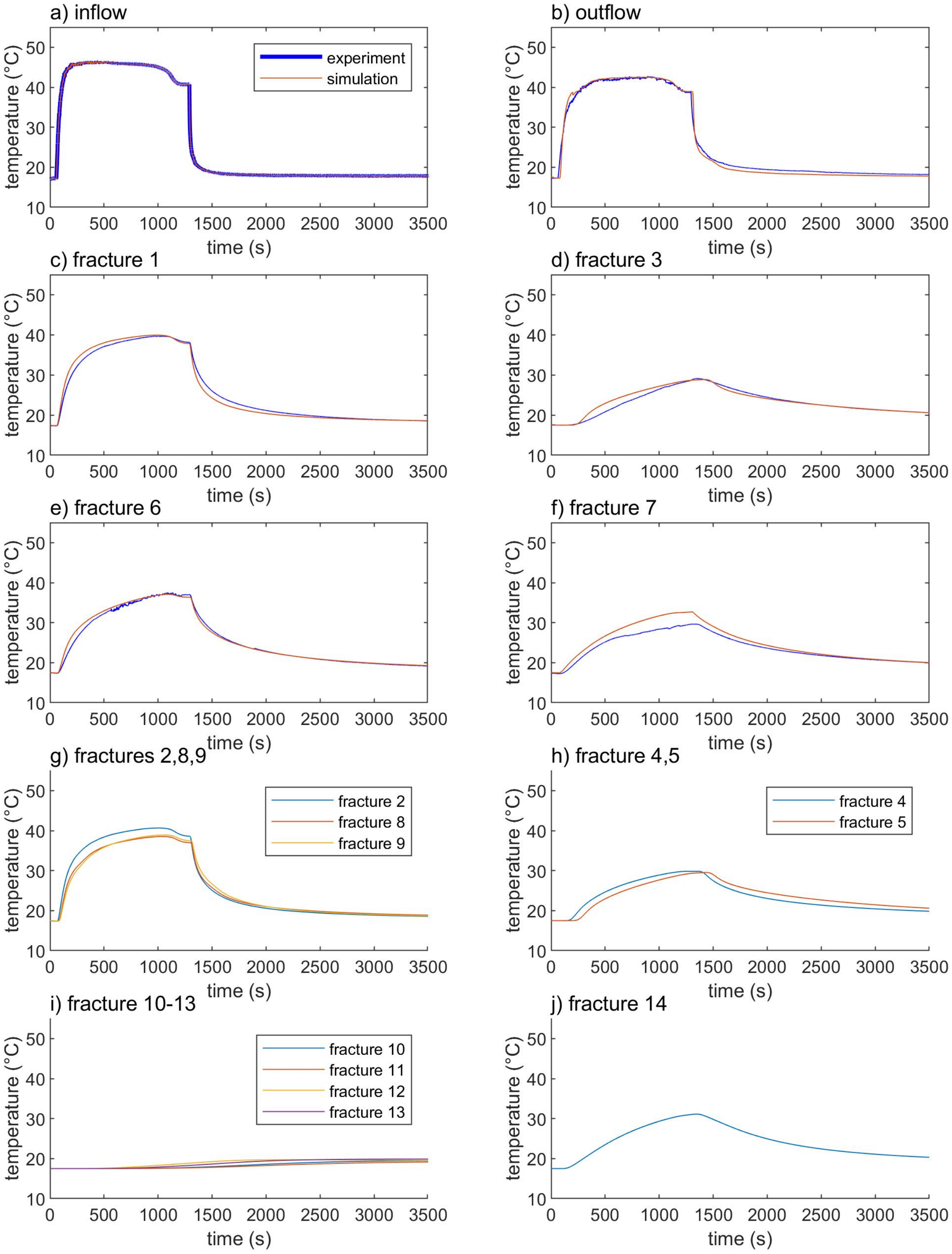

Supplementary Figure 20: Temperatures recorded at six locations in the setup for a flow rate of $8.971 \times 10^{-6}\,\mathrm{m^3\,s^{-1}}$ during the experiment and the numerical simulation at these six locations and in the middle of the remaining fractures.

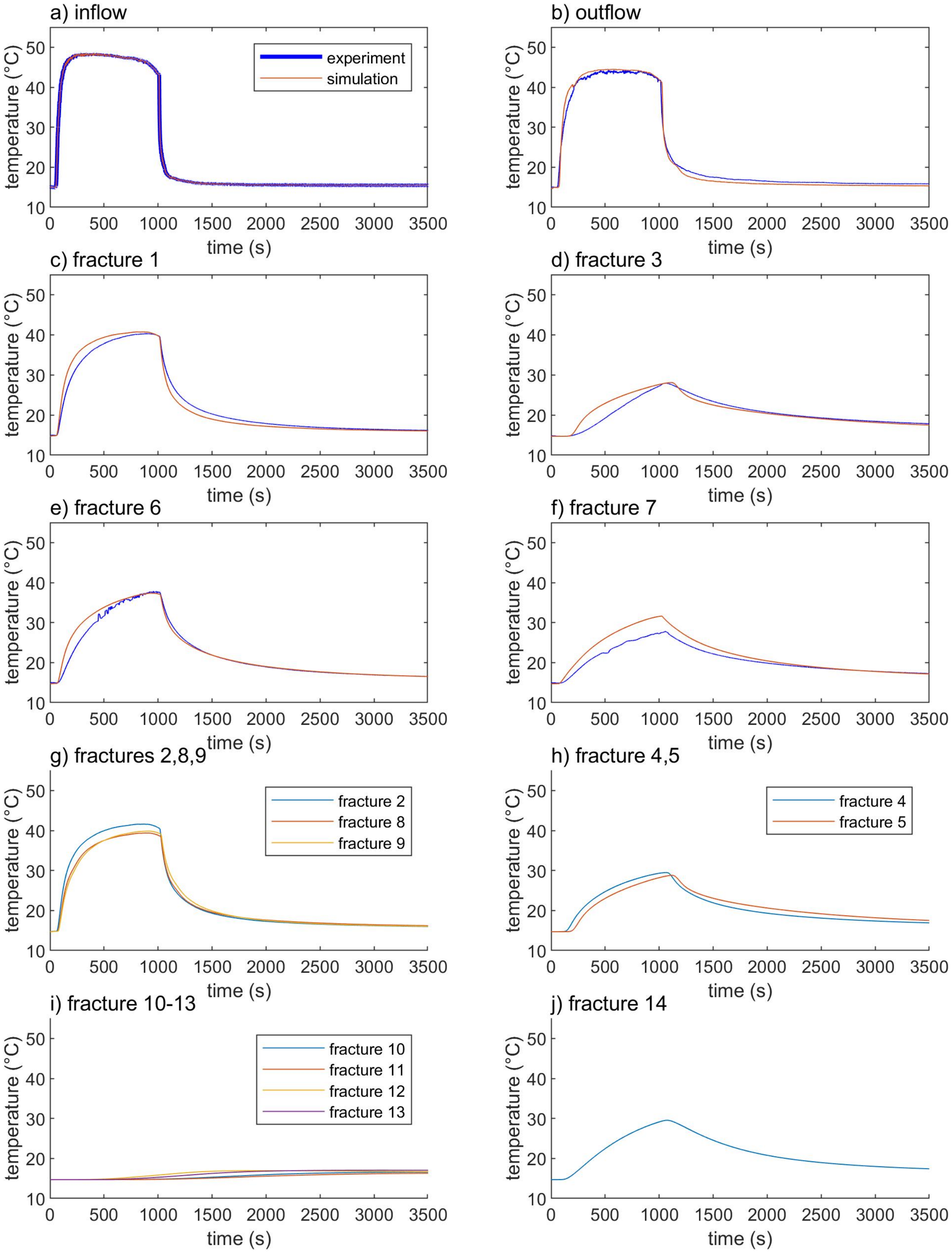

Supplementary Figure 21: Temperatures recorded at six locations in the setup for a flow rate of $1.236 \times 10^{-5}\,\mathrm{m^3\,s^{-1}}$ during the experiment and the numerical simulation at these six locations and in the middle of the remaining fractures.

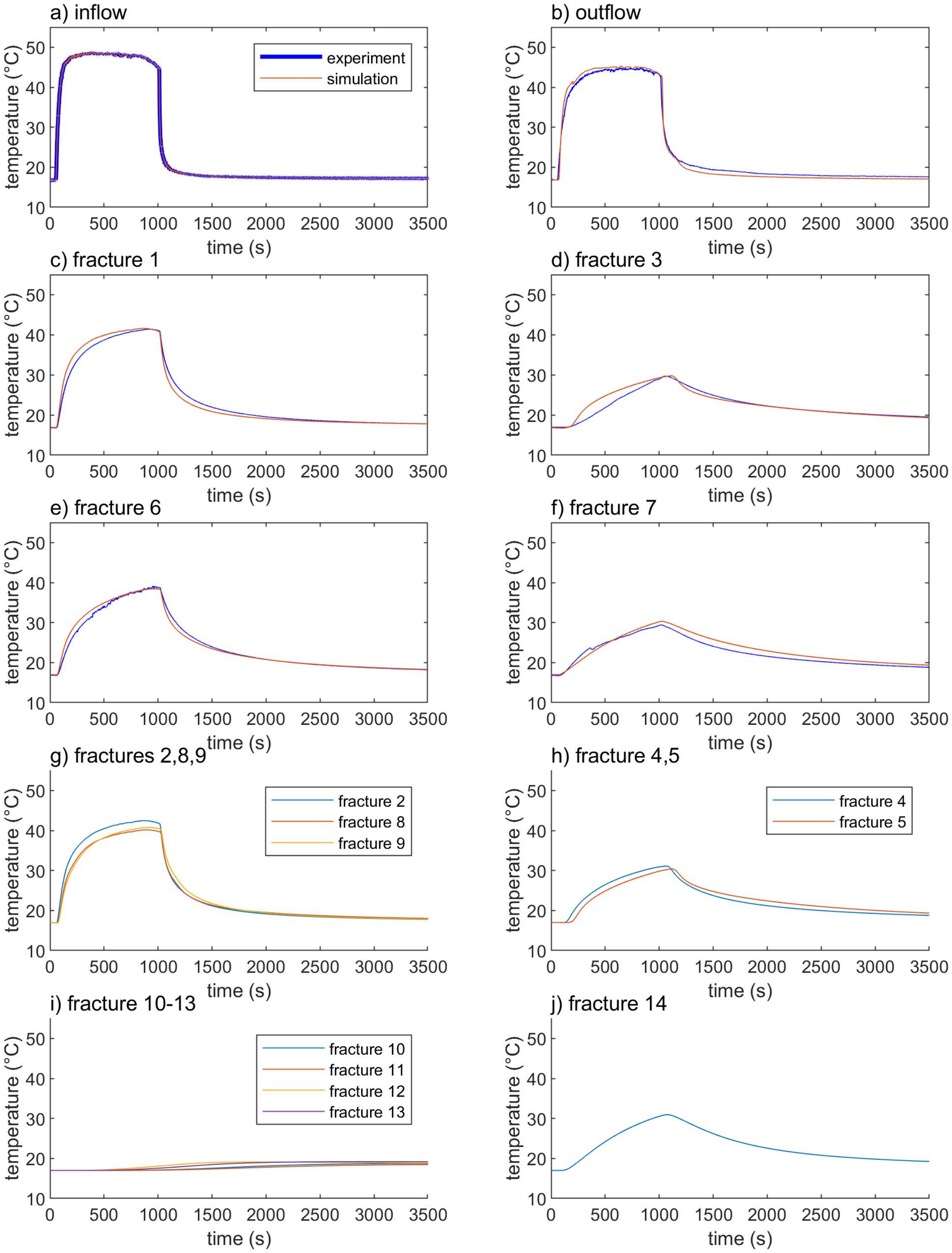

Supplementary Figure 22: Temperatures recorded at six locations in the setup for a flow rate of 1.259×10^{-5} $m^3\,s^{-1}$ during the experiment and the numerical simulation at these six locations and in the middle of the remaining fractures.

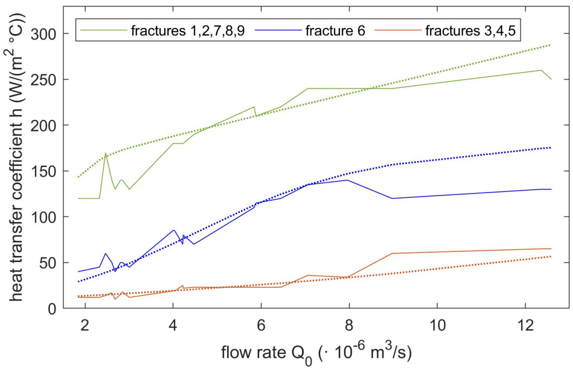

Supplementary Figure 23: Best-fit values for the heat transfer coefficient h (solid lines) and values predicted by the newly presented empirical equation (dashed lines). Separate sets of fractures have different values depending on the flow rate through the respective fractures. The values of h in fractures 10 to 14 is set constant for all flow rates and not shown, as there is no flow in these fractures. All sets of fractures require increasing values for the heat transfer coefficient with increasing flow rate. Outliers at low flow rates are presumably caused by unsteady flow in the network.

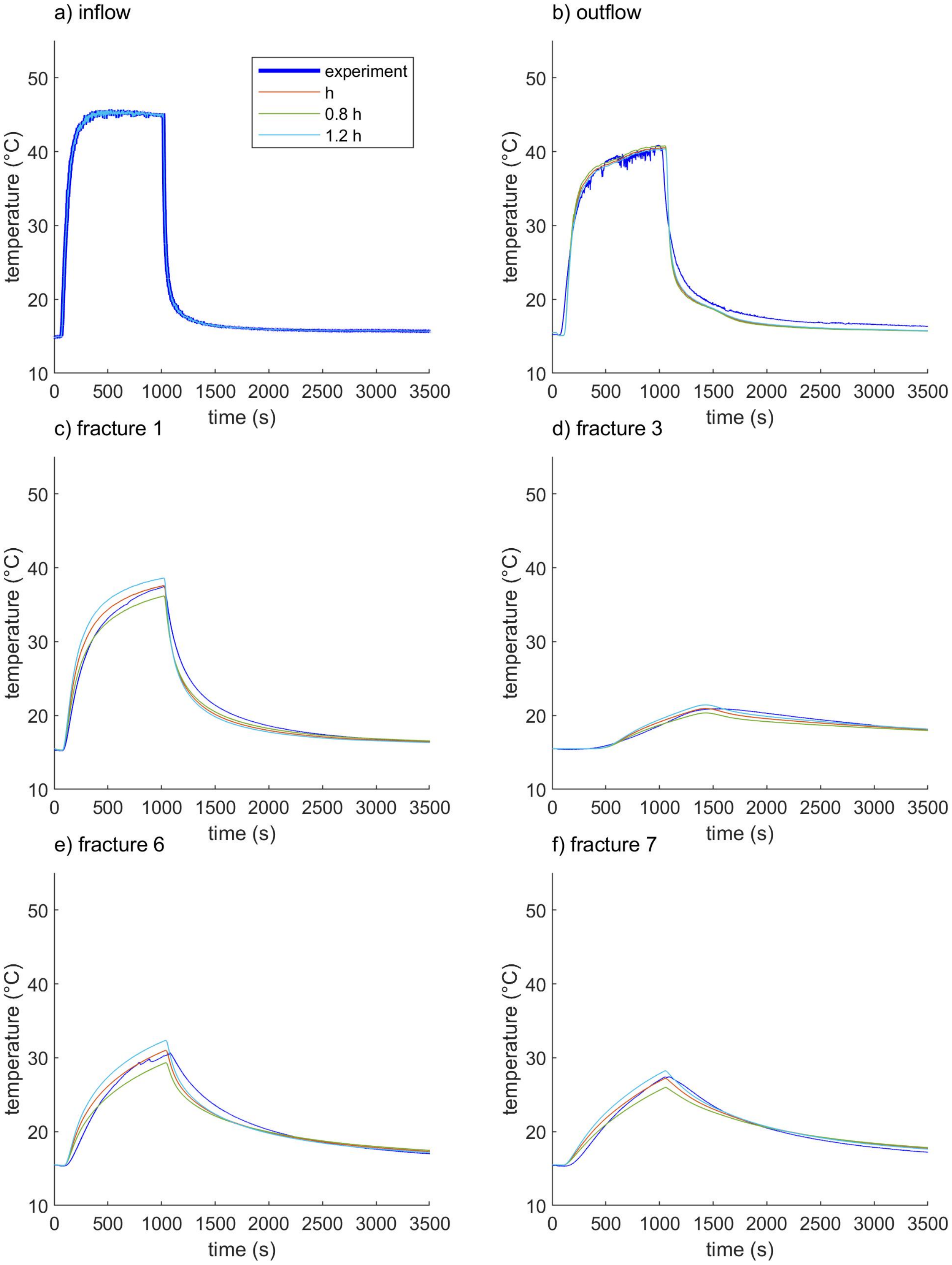

Supplementary Figure 24: Temperatures recorded at six locations in the setup for a flow rate of $4.471 \times 10^{-6}\,\mathrm{m^3\,s^{-1}}$ during the experiment and the numerical simulation at these six locations and in the middle of the remaining fractures. Variation of the best fit value for the heat transfer coefficient by 20 %.

Supplementary Note 2: Reservoir-scale fracture network analysis

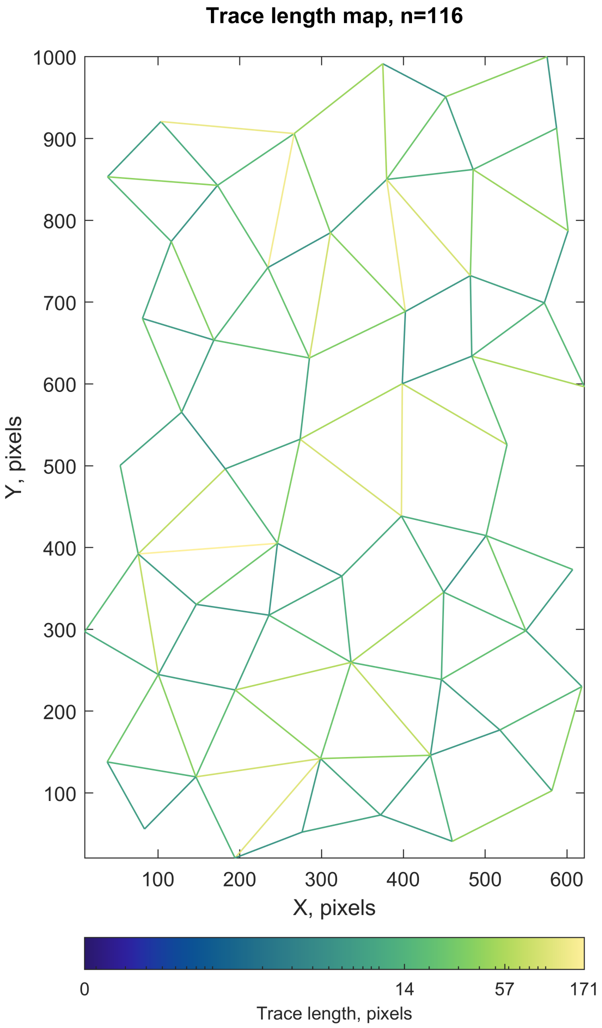

Supplementary Figure 25: Fracture length of the network consisting of 116 fractures analyzed using FracPaQ (Healey at al., 2017: 10.1016/j.jsg.2016.12.003). Pixels equal meters. Line thickness is enhanced for visibility.

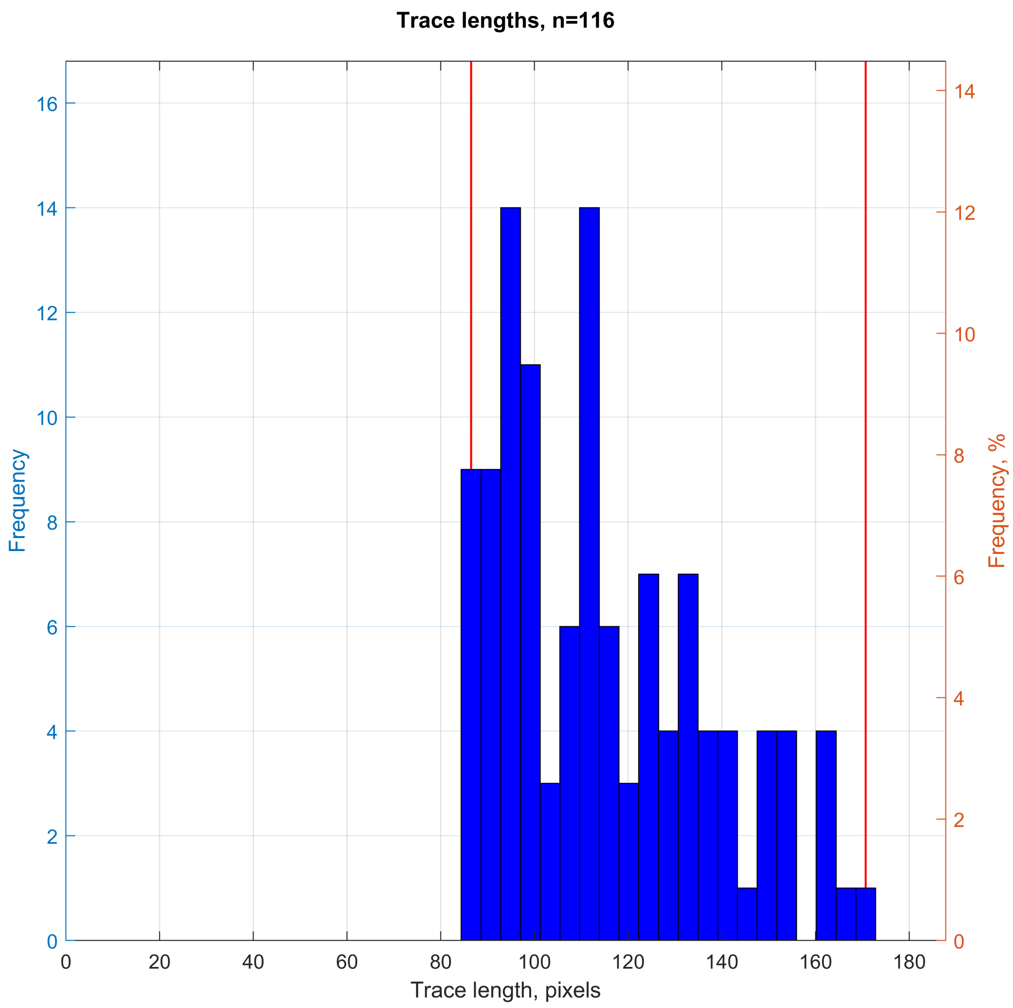

Supplementary Figure 26: Histogram of fracture length of the network consisting of 116 fractures analyzed using FracPaQ. Pixels equal meters.

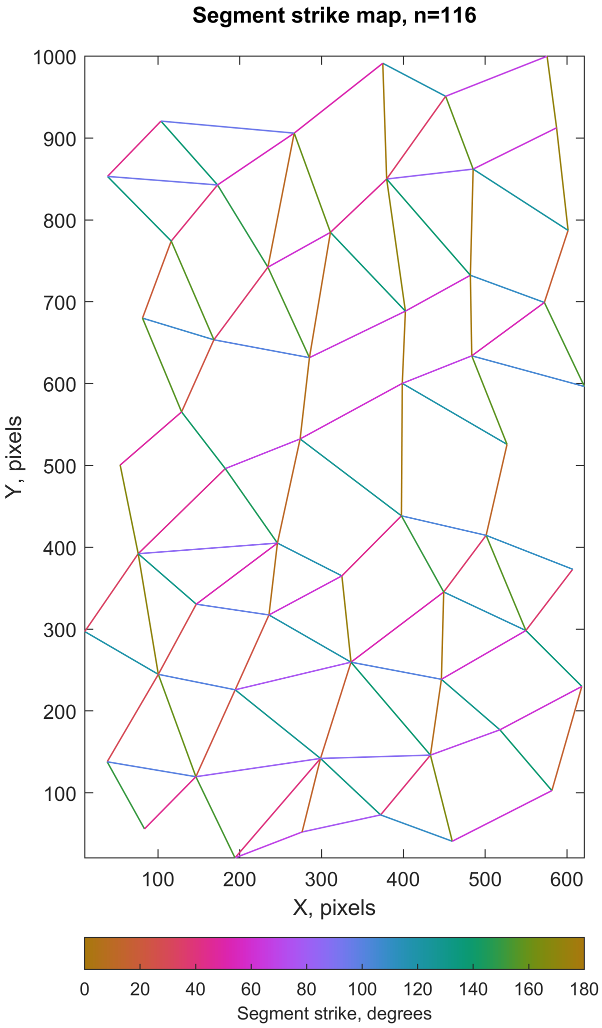

Supplementary Figure 27: Fracture strike of the network consisting of 116 fractures analyzed using FracPaQ. Line thickness is enhanced for visibility.

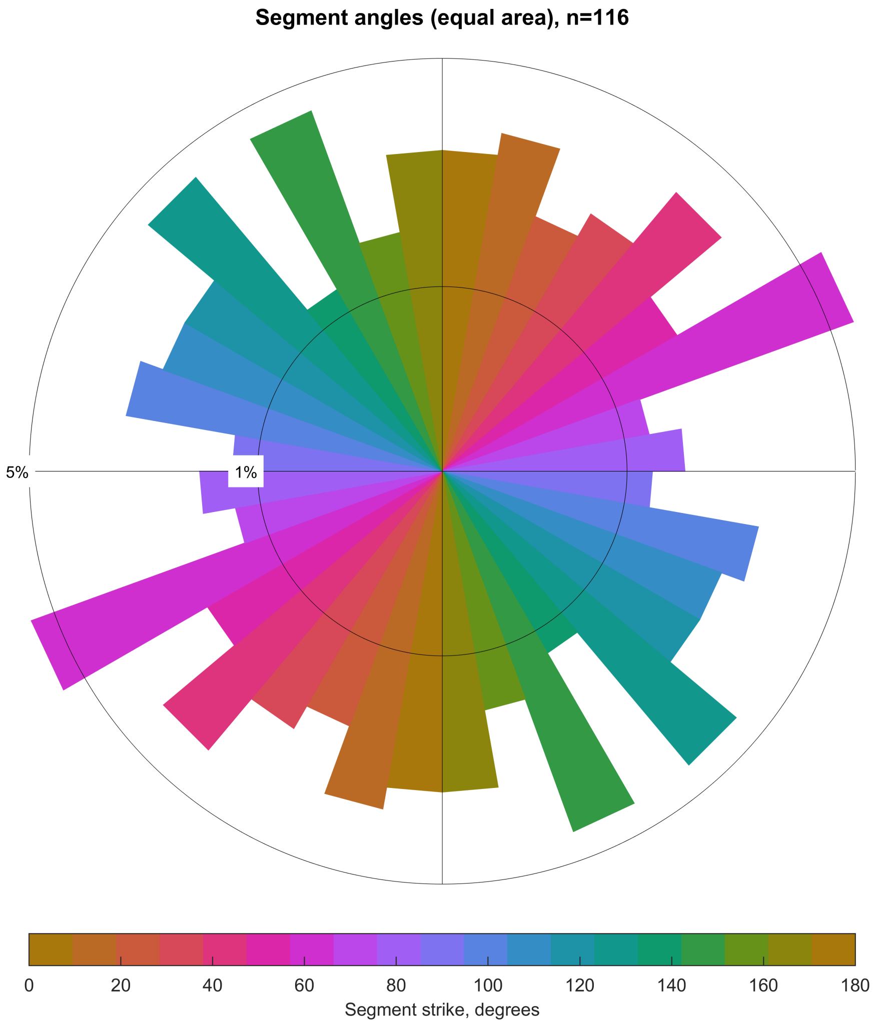

Supplementary Figure 28: Rose diagram of fracture strike of the network consisting of 116 fractures analyzed using FracPaQ.

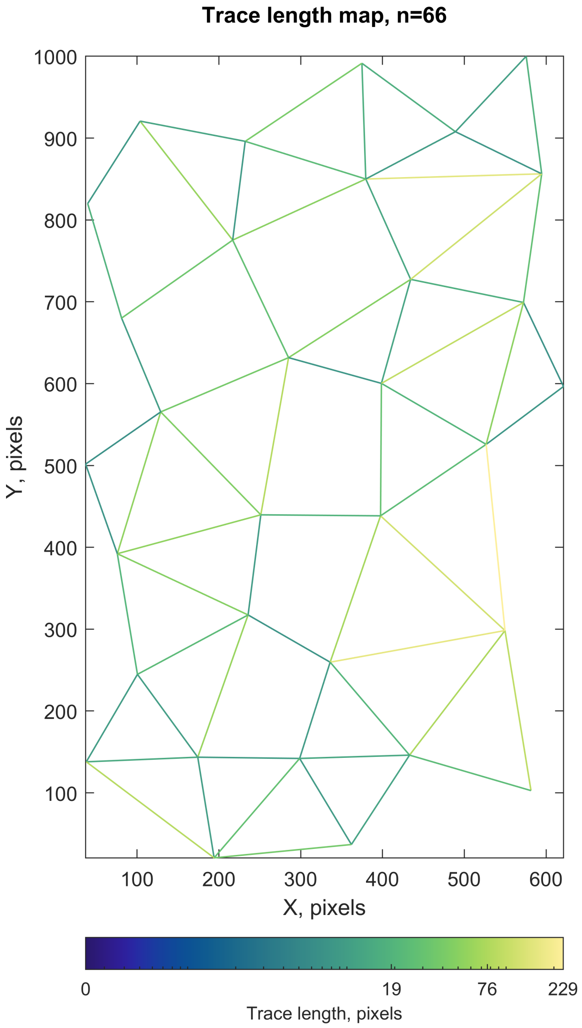

Supplementary Figure 29: Fracture length of the network consisting of 66 fractures analyzed using FracPaQ. Pixels equal meters. Line thickness is enhanced for visibility.

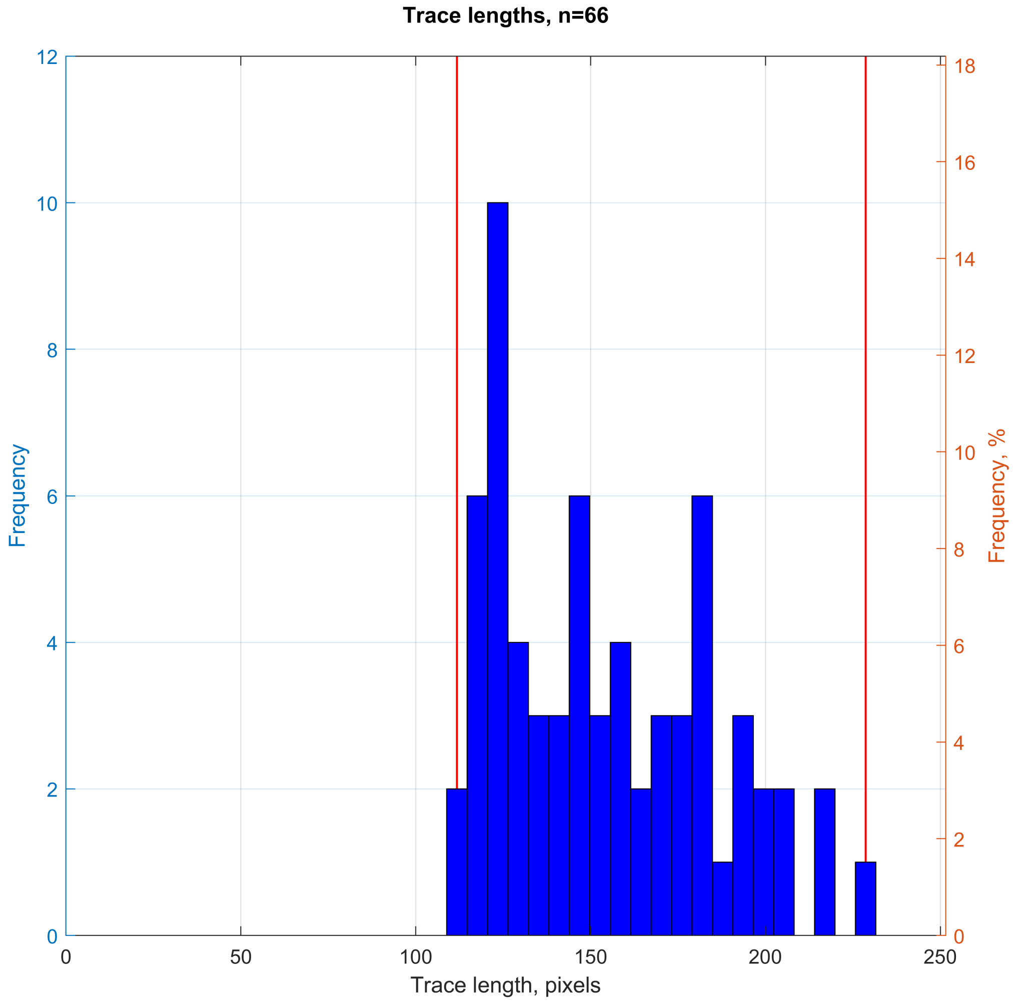

Supplementary Figure 30: Histogram of fracture length of the network consisting of 66 fractures analyzed using FracPaQ. Pixels equal meters.

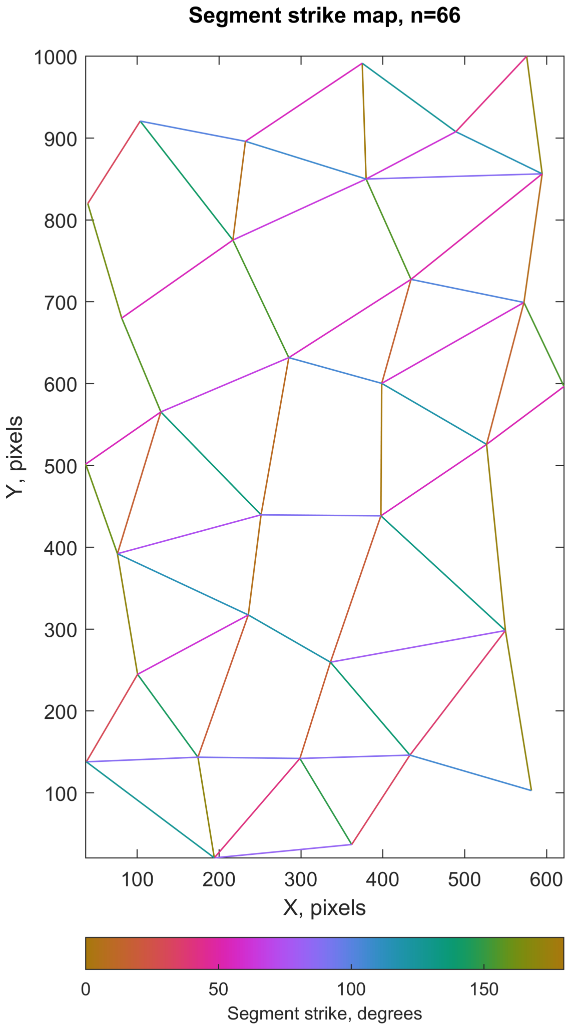

Supplementary Figure 31: Fracture strike of the network consisting of 66 fractures analyzed using FracPaQ. Line thickness is enhanced for visibility.

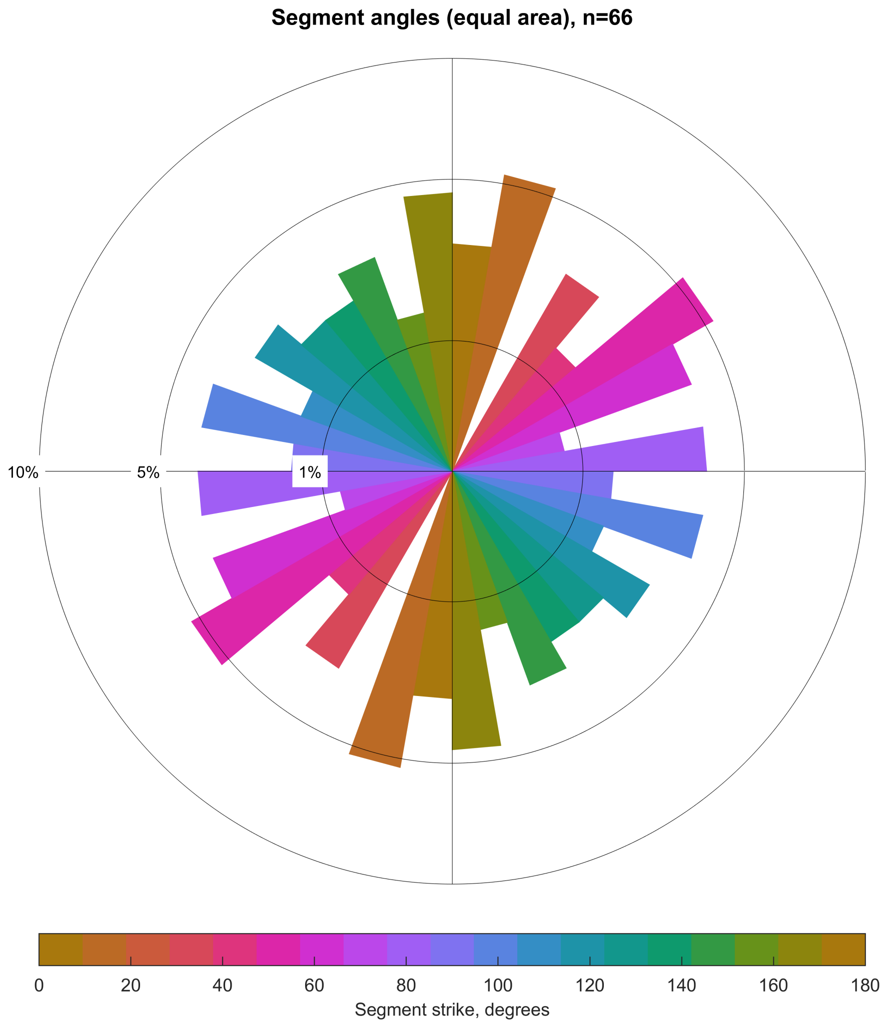

Supplementary Figure 32: Rose diagram of fracture strike of the network consisting of 66 fractures analyzed using FracPaQ.

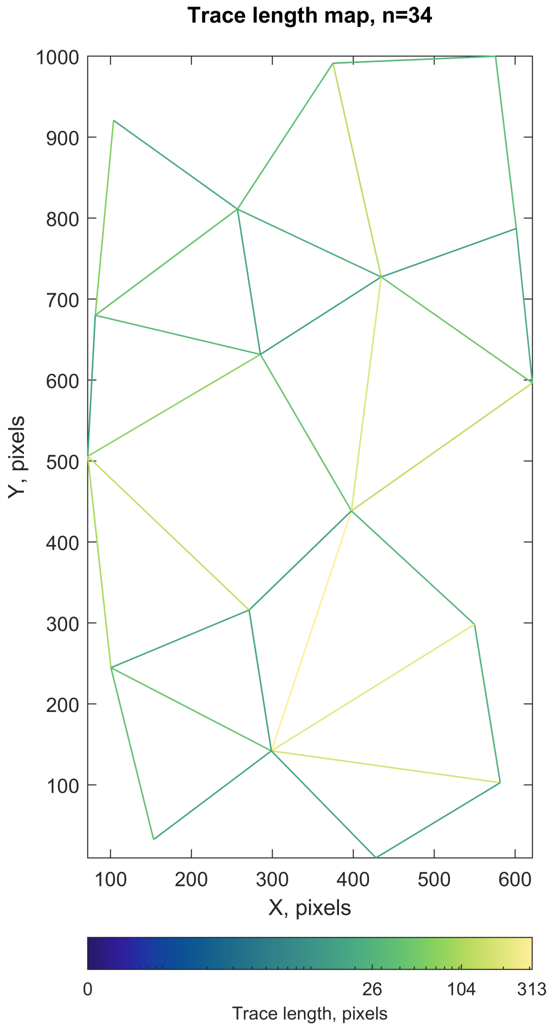

Supplementary Figure 33: Fracture length of the network consisting of 34 fractures analyzed using FracPaQ. Pixels equal meters. Line thickness is enhanced for visibility.

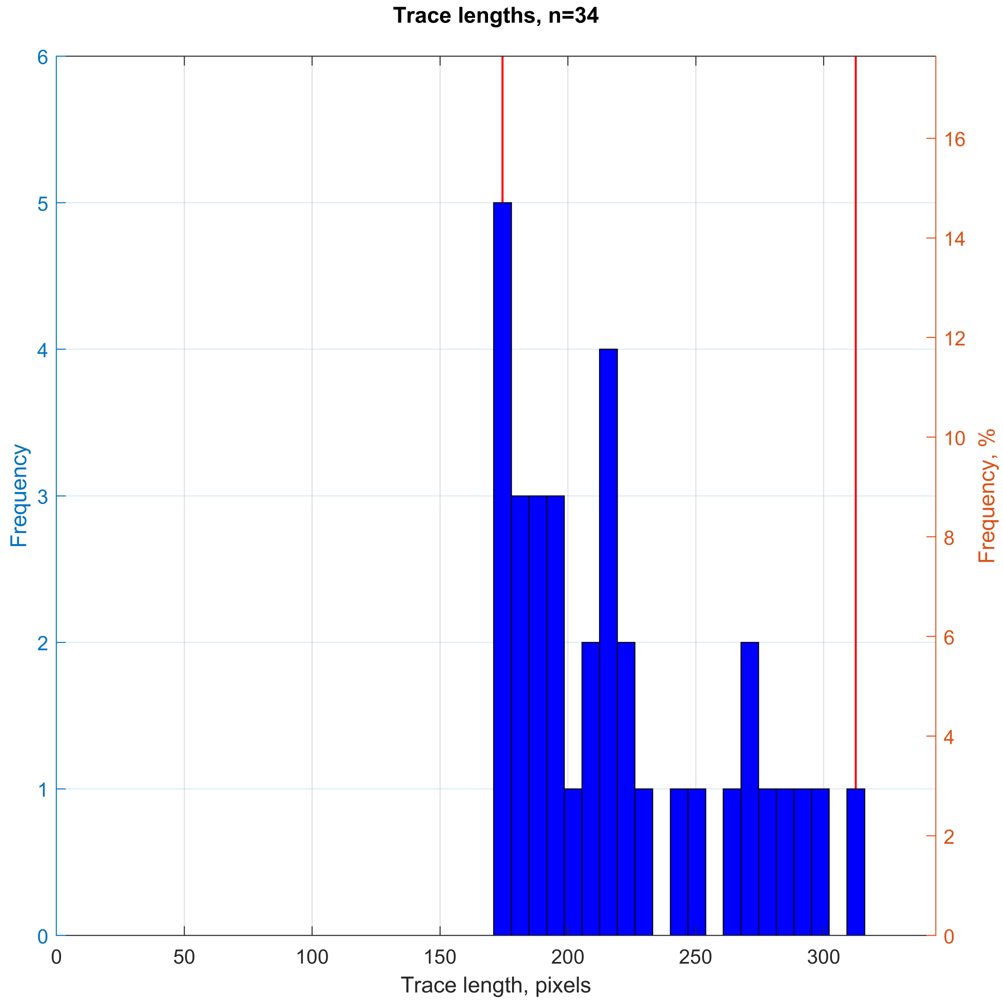

Supplementary Figure 34: Histogram of fracture length of the network consisting of 34 fractures analyzed using FracPaQ. Pixels equal meters.

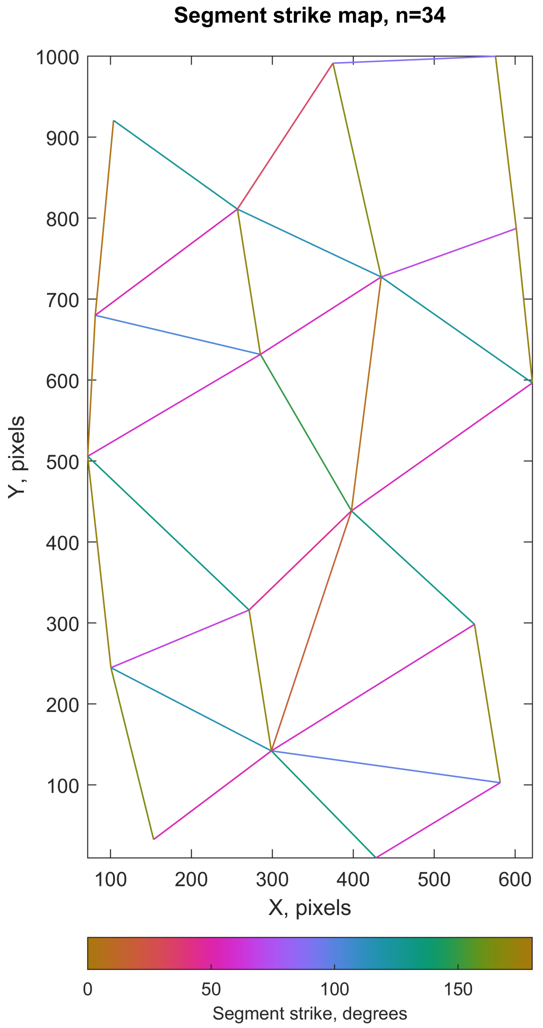

Supplementary Figure 35: Fracture strike of the network consisting of 34 fractures analyzed using FracPaQ. Line thickness is enhanced for visibility.

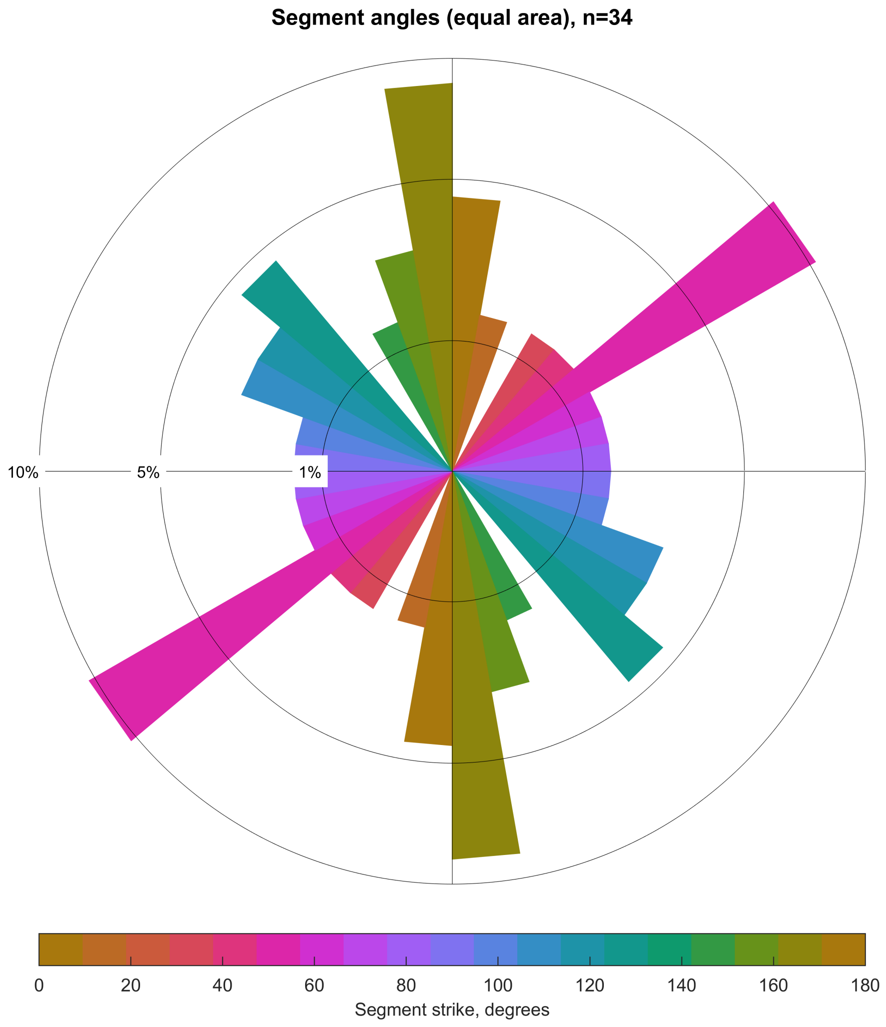

Supplementary Figure 36: Rose diagram of fracture strike of the network consisting of 34 fractures analyzed using FracPaQ.

Supplementary Note 3: Reservoir-scale simulation results

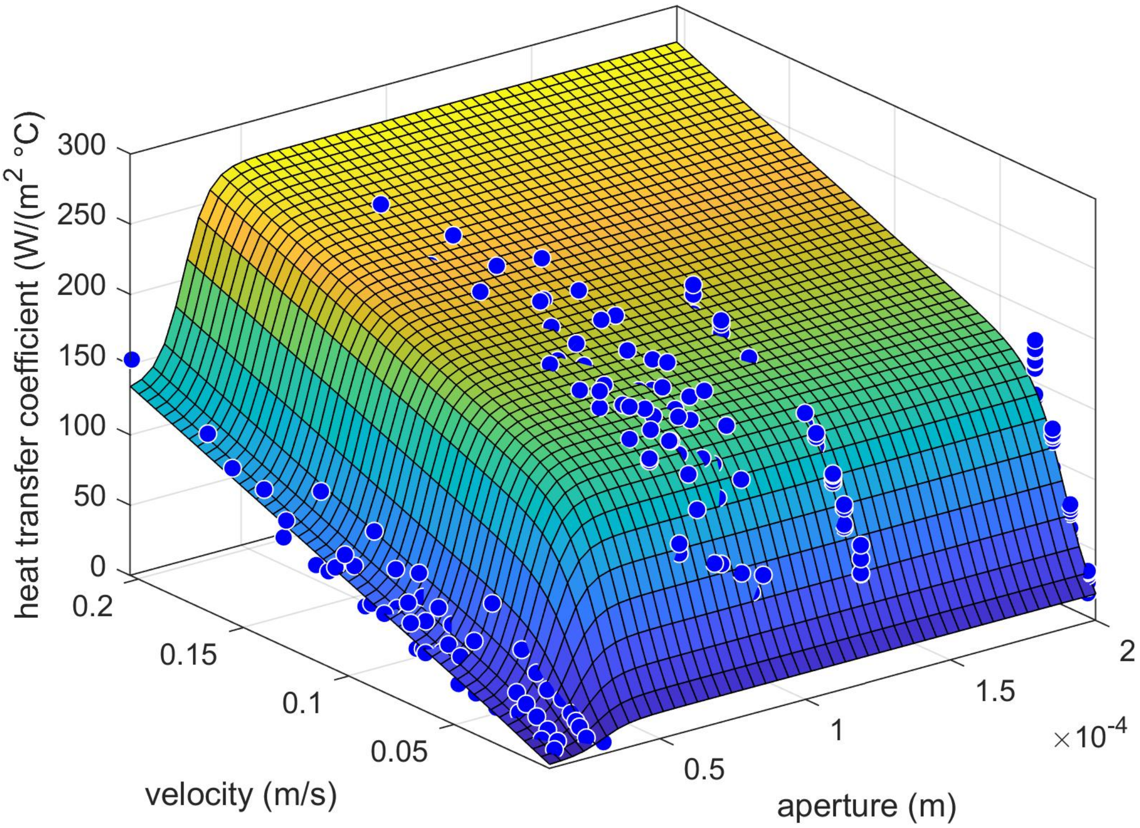

Supplementary Figure 37: Graphical representation of the empirical formula derived based on 240 experiments from [4,17,18] and the analysis of those experiments by [10]

.

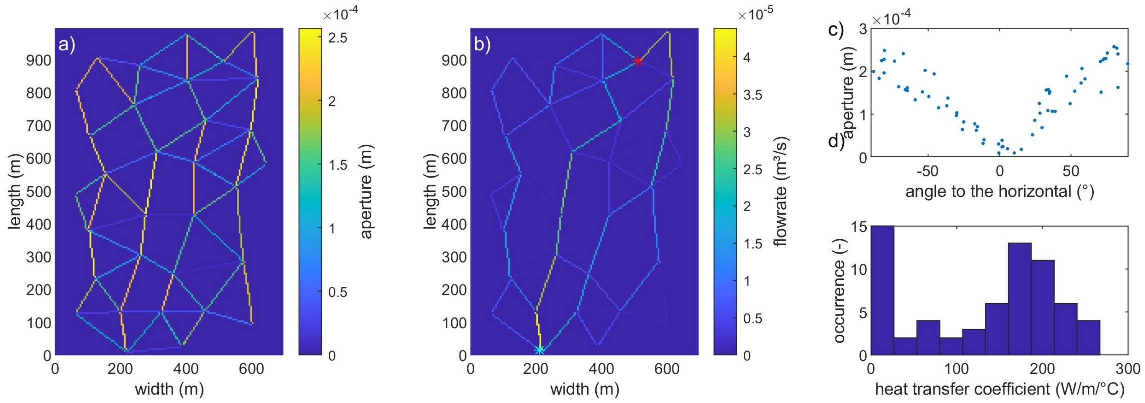

Supplementary Figure 38: Fracture network of the numerical geothermal reservoir with 66 fractures with marked injection (red dot) and production borehole (blue dot). The aperture (a) is calculated based on an assumed stress field oriented roughly north to south (c). The flow rate is maximum along preferential flow paths within the network (b). Line thickness is enhanced for visibility in a & b.

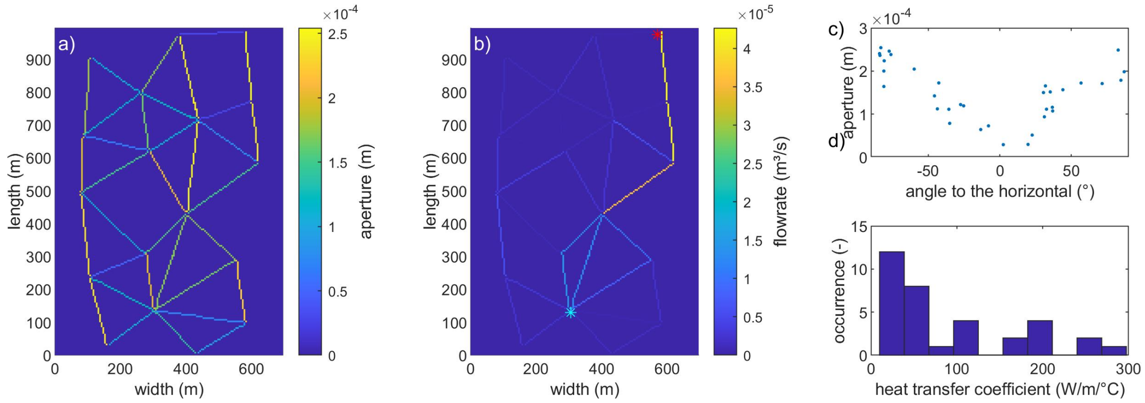

Supplementary Figure 39: Fracture network of the numerical geothermal reservoir with 34 fractures with marked injection (red dot) and production borehole (blue dot). The aperture (a) is calculated based on an assumed stress field oriented roughly north to south (c). The flow rate is maximum along preferential flow paths within the network (b). Line thickness is enhanced for visibility in a & b.

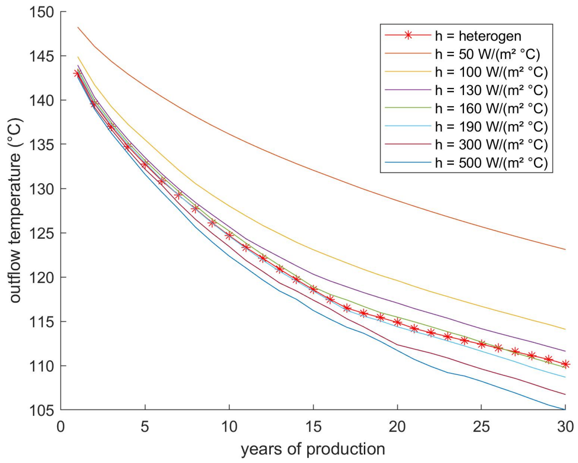

Supplementary Figure 40: Simulated water temperatures at outflow for the network of consisting of 66 fractures for various values of the heat transfer coefficient h equal for all fractures and the heterogeneous velocity dependent approach.

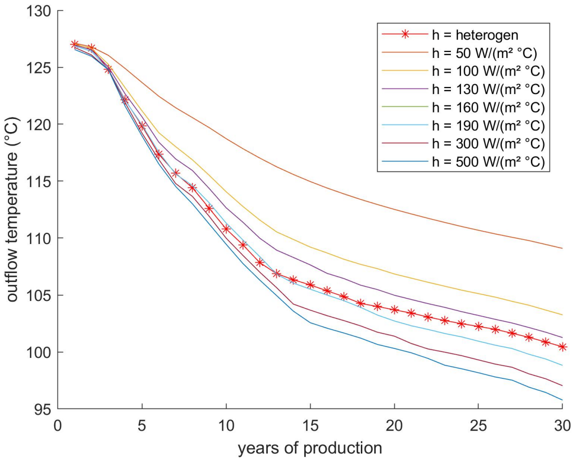

Supplementary Figure 41: Simulated water temperatures at outflow for the network of consisting of 34 fractures for various values of the heat transfer coefficient h equal for all fractures and the heterogeneous velocity dependent approach.

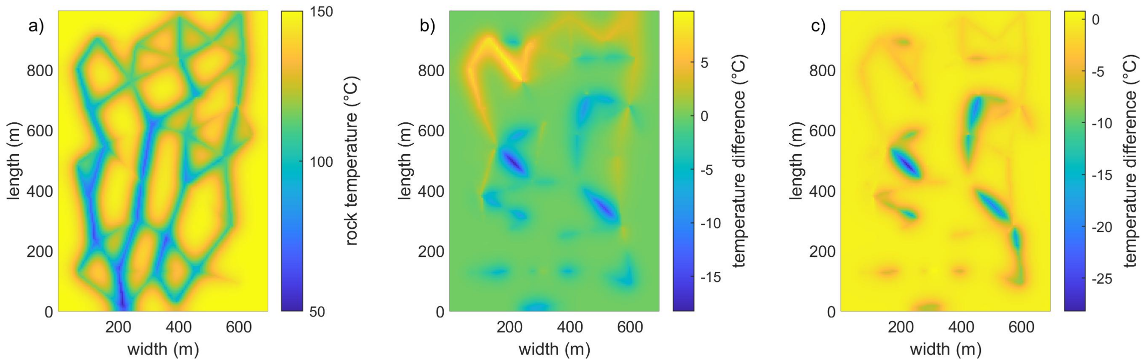

Supplementary Figure 42: Rock temperature for the velocity dependent heat transfer (a) and differences to this distribution for $h = 100\,\mathrm{W\,m^{-2}\,{}^{\circ}C^{-1}}$ (b) and $h = 190\,\mathrm{W\,m^{-2}\,{}^{\circ}C^{-1}}$ (c) in the reservoir consisting 66 fractures. Rock temperature significantly differs around individual fractured as well as for whole sections of the reservoir.

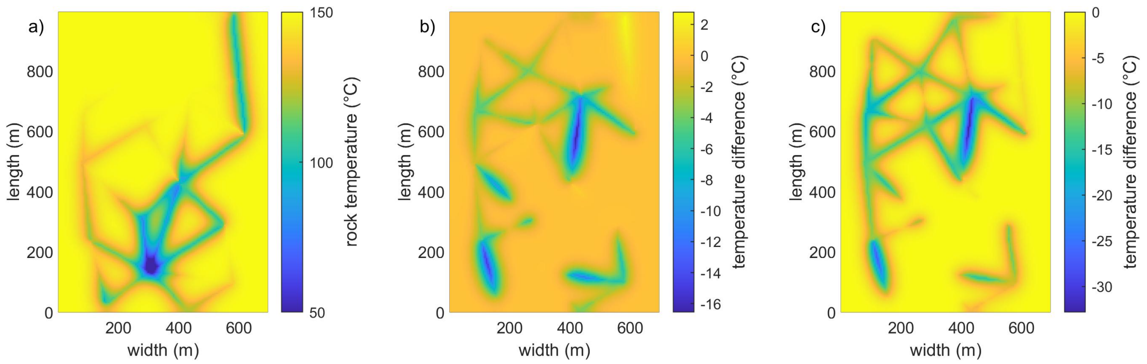

Supplementary Figure 43: Rock temperature for the velocity dependent heat transfer (a) and differences to this distribution for $h = 100\,\mathrm{W\,m^{-2}\,{}^{\circ}C^{-1}}$ (b) and $h = 190\,\mathrm{W\,m^{-2}\,{}^{\circ}C^{-1}}$ (c) in the reservoir consisting of 34 fractures. Rock temperature significantly differs around individual fractured as well as for whole sections of the reservoir.

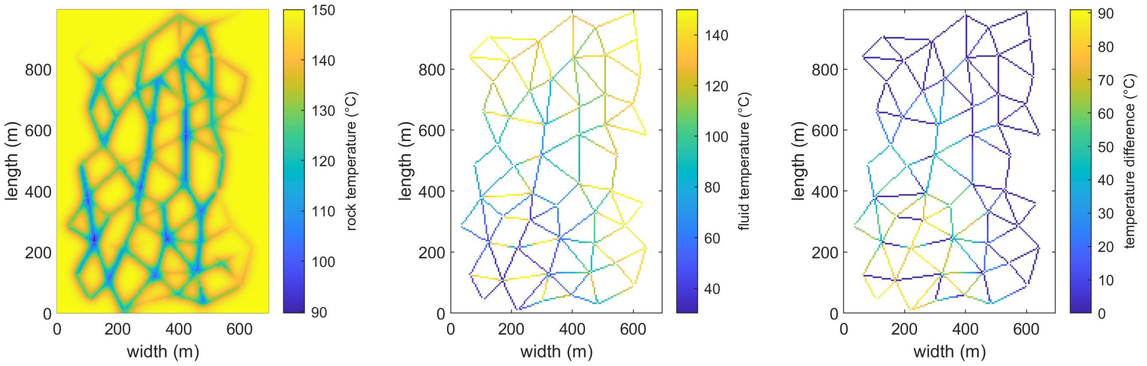

Supplementary Figure 44: Temperature difference between the phases. Rock temperature (a), fluid temperature (b) and the local temperature difference between fluid and rock temperature (c) for the network of 116 fractures after 10 years of production. Fracture thickness (b&c) visually increased for visibility.

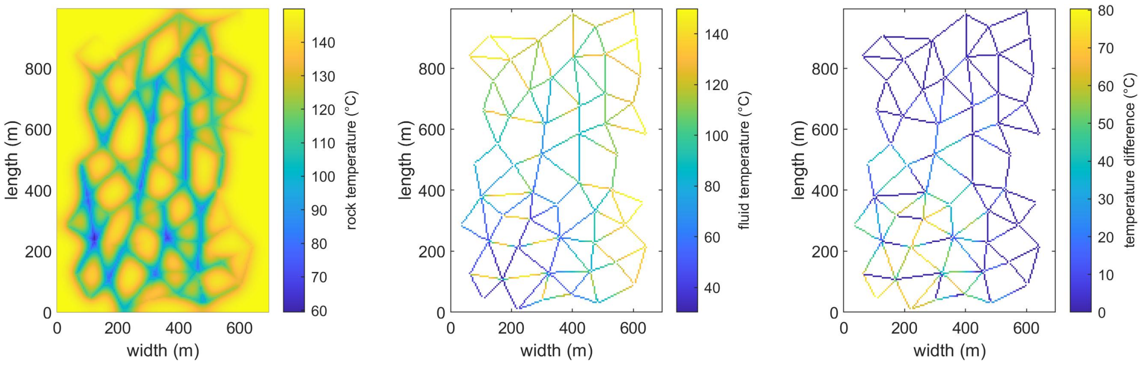

Supplementary Figure 45: Temperature difference between the phases. Rock temperature (a), fluid temperature (b) and the local temperature difference between fluid and rock temperature (c) for the network of 116 fractures after 20 years of production. Fracture thickness (b&c) visually increased for visibility.

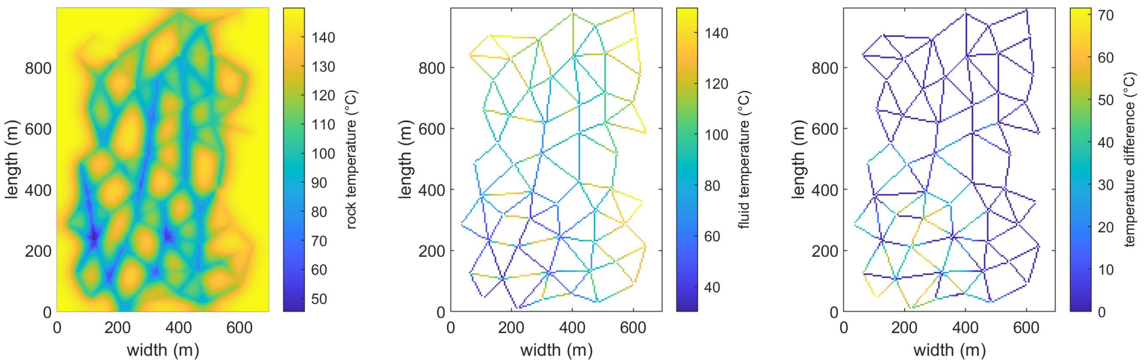

Supplementary Figure 46: Temperature difference between the phases. Rock temperature (a), fluid temperature (b) and the local temperature difference between fluid and rock temperature (c) for the network of 116 fractures after 30 years of production. Over the years, local differences between the phase temperatures declines. Fracture thickness (b&c) visually increased for visibility.

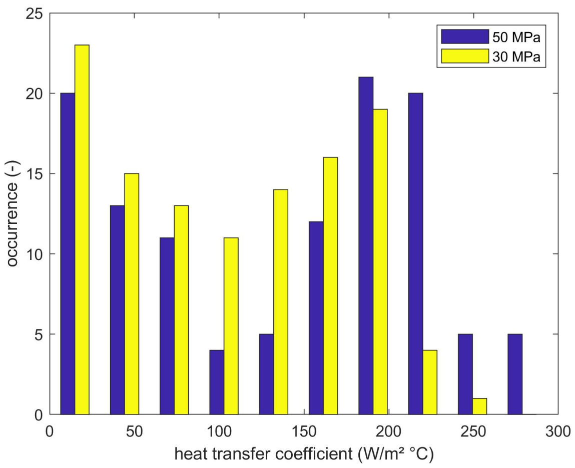

Supplementary Figure 47: Distribution of the heat transfer coefficient for 30 and 50 MPa injection pressure within the reservoir of 116 fractures. Both distributions peak around $190\,\mathrm{W\,m^{-2}\,{}^{\circ}C^{-1}}$.

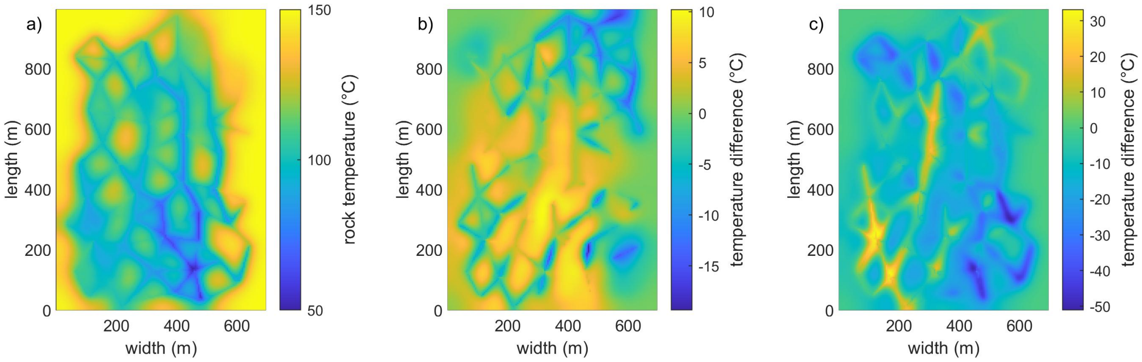

Supplementary Figure 48: Temperature distribution at the end of reservoir lifetime after 53 years of production with the switch from well locations set 1 to well locations set 2 after 33 years for the network with 116 fractures and the velocity dependent heat transfer coefficient (a). Temperature difference to this state for the similar simulation with a homogeneous heat transfer coefficient for $190\,\mathrm{W\,m^{-2}\,{}^{\circ}C^{-1}}$ after 43 years (b) and temperature difference to the temperature distribution after 34 years (c). Parts of the reservoir, especially around the injection borehole of set 1 recover thermally by roughly $30\,{}^{\circ}\mathrm{C}$.
